# Intestinal Microbiota Transplant Prior to Allogeneic Stem Cell Transplant (MAST) trial: study protocol for a multicentre, double-blinded, placebo-controlled, phase IIa trial

**DOI:** 10.1136/bmjopen-2024-093120

**Published:** 2024-12-22

**Authors:** Benjamin H Mullish, Andrew J Innes, Lauren A Roberts, Shian Anim-Burton, Lee Webber, Nicholas A Johnson, Rohma Ghani, Pakhshan Farshi, Anjum B Khan, Francesca Kinsella, Panagiotis Kottaridis, Pramila Krishnamurthy, Emma Nicholson, Renuka Palanicawandar, Graham Wheeler, Frances Davies, Julian R Marchesi, Jiří Pavlů

**Affiliations:** 1 Division of Digestive Diseases, Department of Metabolism, Digestion and Reproduction, Faculty of Medicine, Imperial College London, London, UK; 2 Department of Hepatology, St Mary's Hospital, Imperial College Healthcare NHS Trust, London, UK; 3 Centre for Haematology, Department of immunology and inflammation, Faculty of Medicine, Imperial College London, London, UK; 4 Department of Haematology, Hammersmith Hospital, Imperial College Healthcare NHS Trust, London, UK; 5 Cancer Research UK Imperial Centre, Clinical Trials Section, Department of Surgery and Cancer, Faculty of Medicine, Imperial College London, London, UK; 6 Imperial Clinical Trials Unit, School of Public Health, Faculty of Medicine, Imperial College London, London, UK; 7 Department of Infectious Diseases, Hammersmith Hospital, Imperial College Healthcare NHS Trust, London, UK; 8 Department of Haematology, Manchester Royal Infirmary, Manchester, UK; 9 Department of Haematology, Leeds Teaching Hospitals NHS Trust, Leeds, UK; 10 Department of Haematology, University Hospitals Birmingham NHS Foundation Trust, Birmingham, UK; 11 Department of Haematology, University College London Hospitals NHS Foundation Trust, London, UK; 12 Department of Haematology, King's College Hospital NHS Foundation Trust, London, UK; 13 Department of Haematology, The Royal Marsden Hospital, London, UK; 14 Statistics and Data Science Innovation Hub, GSK, London, UK

**Keywords:** Bone marrow transplantation, Leukaemia, Transplant medicine, Microbiota

## Abstract

**Introduction:**

Lower diversity of the gut microbiome prior to allogeneic haematopoietic cell transplantation (HCT) correlates with reduced survival after the intervention. Most patients undergoing HCT for a haematological malignancy have previously received intensive chemotherapy, resulting in prolonged neutropenic episodes requiring broad-spectrum antibiotics; use of these has been linked to reduced microbiome diversity. Intestinal microbiota transplant (IMT) is a novel treatment approach that restores this diversity. We hypothesised that IMT performed prior to initiation of HCT conditioning restores microbiome diversity during the early stages of HCT, leading to decreased frequency of complications and improved outcomes of HCT.

**Methods and analysis:**

50 adult patients receiving allogeneic HCT will be recruited into this phase IIa trial and randomised 1:1 to receive capsulised IMT or matched placebo shortly prior to initiation of HCT conditioning and followed for up to 12 months. The primary outcome will be to assess the increase in alpha diversity between pre-IMT and that measured at ~42 days after IMT administration (day +28 of HCT), comparing the difference between patients receiving IMT compared with placebo. Secondary outcomes will include tolerability, the dynamics of gut microbiome diversity metrics and taxonomy over all time points assessed, as well as clinical outcomes (including burden of invasive infections, days of fever, admission to intensive care, development of graft-vs-host disease and mortality).

**Ethics and dissemination:**

This study was approved by a UK Research Ethics Committee (REC reference: 23/NE/0105). Dissemination of results will be in concert with patient and public involvement group input and is expected to be primarily via abstract presentation at conferences and manuscripts in peer-reviewed journals.

**Trial registration numbers:**

NCT6355583; EudraCT: 2022-003617-10.

**STRENGTHS AND LIMITATIONS OF THIS STUDY**

- While our prior observational study of intestinal microbiota transplant (IMT) administered prior to haematopoietic cell transplantation (HCT) suggested clinical benefits, the multicentre, randomised, placebo-controlled nature of the Microbiota Transplant Prior to Allogeneic Stem Cell Transplant trial will enable exploration of this observation in a more robust setting.
- This study will use a multidonor capsule IMT preparation (as opposed to conventional IMT slurry), which was the patient preference during our patient and public involvement group feedback, primarily due to its less invasive nature.
- However, we have no prior ‘head to head’ testing of IMT slurry compared with this capsule preparation in terms of efficacy.
- The timing of IMT pre-HCT (to ‘prehabilitate’ the gut microbiome) is a distinctive novel aspect of this study—there is a biological rationale to support this choice, but there are certain drawbacks of this approach too.
- Together with recording clinical outcomes post-IMT and HCT, we will collect patient samples for immunological and multiomic profiling (including microbiome and metabolome analysis), to better understand the mechanisms of action of IMT in this setting.

## Introduction

 Allogeneic haematopoietic cell transplantation (HCT) is a powerful therapeutic modality for patients with acute leukaemia (AL) and certain other haematological malignancies. Furthermore, with the advent of reduced intensity protocols and approaches that permit safer use of mismatched donors, its frequency is increasing. In preparation for transplant, patients receive a conditioning regimen of high-dose chemotherapy and/or total-body irradiation, followed by infusion of compatible haematopoietic cells; the engraftment of these cells restores the recipient’s haematopoesis and exerts long-term remission from the graft-versus-leukaemia effect of the donor immune effector cells. However, this process is associated with marked perturbation of the gut microbiome, including reduced gut barrier integrity, loss of gut microbiome diversity and microbiome enrichment in pathobiont bacteria.^1 2^ The immunosuppressive nature of both the underlying haematological malignancy, together with the treatments used, collectively result in a markedly increased risk of infections in these patients. More specifically, the increase in susceptibility to infection leads to an increase in antibiotic exposure, driving the overgrowth of pathobionts and further selection pressure for dominance by antimicrobial resistance genes in the gut.^3^ In this setting, the impact of antibiotics and multidrug-resistant organism (MDRO)-associated infection is associated with poorer clinical outcomes in patients^4^; for example, the use of imipenem-cilastatin or piperacillin-tazobactam use to treat neutropenic fever has been associated with increased graft-versus-host disease (GvHD) mortality up to even 5 years post-HCT.^5^

A large observational study of more than 1300 patients from 4 centres observed that patients with patterns of gut microbiota disruption characterised by loss of diversity had a higher risk of transplantation-related death and death attributable to GvHD.^6^ Baseline samples obtained before HCT already showed evidence of gut microbiome disruption, and lower diversity before transplantation was closely associated with poor survival. Specific gut taxonomic features have also been linked with allo-HCT outcome; specifically, expansion of *Enterococcus* (particularly *Enterococcus faecium*) was observed across the period of having allo-HCT. *Enterococcus* was found to associate closely with GvHD and mortality, with the presence of the disaccharide lactose identified as a factor that promoted *Enterococcus* expansion.^7^

These data support the hypothesis that a microbiome-based intervention, performed prior to initiation of HCT conditioning, may improve microbiome diversity during transplant and has the potential to impact on clinical outcomes. Several approaches have been considered in this setting,^8^ including dietary/prebiotic interventions, probiotics and non-absorbable antibiotics (such as rifaximin). In this study, we have opted for a biological approach that attempts to restore the whole gut ecosystem, using intestinal microbiota transplant (IMT; also known as ‘faecal microbiota transplant’). IMT consists of transferring minimally processed stool, derived from a healthy screened donor, into the gut of a recipient. This approach was pioneered in patients with recurrent *Clostridioides difficile* infection (rCDI), in which the major risk factor is recurrent antibiotic exposure.^9^ The success in this setting has led to the exploration of IMT in other conditions which the gut microbiome appears contributory to their aetiopathogenesis,^10^ with promising early data. Despite initial concerns about safety of IMT in immunocompromised patients—driven in part by descriptions of pathogen transmission via IMT in such patients^11^—adherence to strict screening protocols results in a safety profile comparable to that in immunocompetent recipients.^8 9 12^

After previous reports that the use of IMT for rCDI was also associated with reduced antibiotic resistance genes within the gut microbiome,^13^ and our own observation of a clinical case where IMT seemed to show clinical benefit when used prior to HCT in a patient colonised with MDROs,^14^ we completed a cohort study of IMT performed prior to initiation of HCT conditioning in patients colonised and/or previous infected with MDROs. While we observed that rates of decolonisation of intestinal MDROs were comparable to that observed spontaneously, we saw a significant reduction in rates of bloodstream infection (including MDRO related), length of stay and days of carbapenem use, compared with a matched historical control arm.^15^ With longer follow-up, these benefits translated to improvement in overall survival, such that the poor outcome associated with MDRO colonisation could be negated with IMT.^16^

In this clinical trial, we will investigate the broader role of IMT in HCT, both with and without MDRO colonisation. By randomising patients to receive IMT or placebo prior to HCT conditioning and measuring microbiota diversity, in stool, as a surrogate for its impact on gut ecology, we will determine the capacity of capsule IMT to restore a normal microbiome and track the impact during HCT. Using multiomic profiling of stool, urine and blood, we will investigate the wider impact of IMT in HCT patients, while clinical parameters will explore the potential to impact overall outcome.

## Methods and analysis

### Design and objectives

The Microbiota Transplant Prior to Allogeneic Stem Cell Transplant (MAST) study is a multicentre, randomised, phase IIa double-blind placebo-controlled trial. The major objective of this trial is to determine the ability of capsulised IMT given prior to allogeneic HCT to increase and maintain stool microbiota diversity after HCT from baseline. Secondary objectives include determination of clinical tolerability, effects of capsule IMT on clinical outcomes and to explore microbiome and immune dynamics related to IMT use. The primary outcome is the change in alpha diversity (measured as inverse Simpson’s index) after IMT administration measured immediately prior to IMT (at 14±3 days prior to HCT) and 28±3 days after HCT, comparing the change between patients receiving capsulised IMT versus placebo. The study is sponsored by Imperial College London. This is an investigator-led study; while funding for the study was only awarded after peer review, the funder, sponsor and industrial partner have had no direct role in any aspect of study design (although the funder arranged external peer review as part of the process to the award of funding, which did impact on study design). We have used the Standard Protocol Items: Recommendations for Interventional Trials (SPIRIT) checklist in writing this report.^17^

The study start date was April 2024, with primary completion estimated as August 2026 and study completion estimated as March 2027.

### Recruitment strategy

#### Site selection

We have partnered with several of the largest haematology centres in the UK as recruitment sites to support adequate participant enrolment. These centres are the Royal Marsden Hospital, University College London Hospital, King’s College London Hospital, University Hospital Birmingham, Leeds Teaching Hospital, Hammersmith Hospital, and Manchester University Hospital. Such centres are well positioned to provide access to a high volume of eligible patients, due to their expertise and patient population in haematology and transplant services.

#### Engagement with patient advocacy groups

From its inception, the MAST trial was codeveloped with a patient and public involvement (PPI) group, itself based around the NCRI (National Cancer Research Institute) acute myeloid leukaemia (AML) Supportive care group. The group refined the protocol and participant-facing documents and provided input into the design to improve the communication and reach of the study to potential participants.

#### Regular communication and updates

Our dedicated trials unit maintains regular communication with participating sites to support recruitment efforts. This includes helping to support barriers to enrolment and providing ongoing assistance to sustain recruitment momentum, ensuring that sites have the resources and support needed to meet target enrolment goals.

#### Patient support and accessibility measures

We have developed study several supportive resources to improve participant understanding, engagement and accessibility to help boost retention. Examples include informational videos to guide participants providing samples, study-specific standard operation procedures to streamline processes across sites and maintain consistency and translated versions of the participant information sheets (PISs) to accommodate diverse language needs. Additionally, funding for transportation costs is available to reduce transportation barriers to make participation more accessible.

### Study setting and participants

This trial will be performed across seven Haematology Units in the UK which regularly undertake HCT. The study will recruit adults with acute lymphoblastic leukaemia (ALL), AML, AL of ambiguous lineage, high-risk myelodysplastic syndrome (MDS), chronic myelomonocytic leukaemia (CMML) and chronic myeloid leukaemia (CML) in blast phase, considered suitable/fit for allogeneic HCT. Patients will be eligible to enter the study if they achieved complete remission (defined as <5% blasts), have received a minimum of two cycles of intensive chemotherapy (online supplemental material 1, appendix 1) and have received broad-spectrum antibiotics within 3 months of HCT. Inclusion and exclusion criteria are summarised below:

#### Inclusion criteria

1. Patients aged 18 years and over with a morphological documented diagnosis of ALL, AML, AL of ambiguous lineage, MDS, CMML and CML in blast phase (online supplemental material 1, appendix 2) who are deemed fit for allogenic HCT with one of the following disease characteristics:

ALL, AML, AL of ambiguous lineage

- Patients in first complete remission (CR1) or second complete remission (CR2) including complete remission with incomplete blood count recovery with <5% blasts (online supplemental material 1, appendix 2).
- Secondary leukaemia (defined as a history of MDS, antecedent haematological disease or chemotherapy exposure) in CR1 or CR2 defined as <5% blasts (online supplemental material 1, appendix 2).

MDS and CMML

- Patients with advanced or high-risk MDS with an IPSS-M moderate high or higher including intermediate or high-risk CMML who have <5% blasts at the time of randomisation (online supplemental material 1, appendix 2).

CML in blast phase

- Patients with Philadelphia or BCR:ABL1 positive CML in blast phase defined by the presence of ≥20% blasts in blood or bone marrow who have achieved second chronic phase with <5% blasts (online supplemental material 1, appendix 2).

1. Patients must have completed minimum of two cycles of intensive chemotherapy prior to trial enrolment (online supplemental material 1, appendix 1).
2. Patients must have received broad-spectrum antibiotics within 3 months prior to trial enrolment.
3. Patients must be considered suitable/fit to undergo allogeneic HCT, as clinically judged by the local investigator.
4. Patients with a Karnofsky Performance Status score 60 or above (online supplemental material 1, appendix 3).
5. Females and male patients of reproductive potential (ie, not postmenopausal or surgically sterilised) must use appropriate, highly effective, contraception from the point of commencing therapy until 6 months after treatment.
6. Patients have given written informed consent.
7. Patients willing and able to comply with scheduled study visits and laboratory tests.

#### Exclusion criteria

1. Patients with contraindications to receiving allogeneic HCT.
2. Female patients who are pregnant or breast feeding. All women of childbearing potential must have a negative pregnancy test before commencing treatment.
3. Adults of reproductive potential are not willing to use appropriate, highly effective, contraception during the specified period.
4. Patients with renal or hepatic impairment as clinically judged by the local investigator.
5. Patients with active infection, HIV-positive, and/ or with chronic active HBV (hepatitis B virus) or HCV (hepatitis C virus) infection.
6. Patients with a concurrent active malignancy or a prior malignancy, except lobular breast carcinoma in situ, fully resected basal cell or squamous cell carcinoma of skin or treated cervical carcinoma in situ, incidental histological finding of prostate cancer (T1a or T1b using the tumour, node, metastasis clinical staging system), previous MDS, CMML, MPN resulting in secondary AML. Cancer treated with curative intent ≥5 years previously will be allowed. Cancer treated with curative intent <5 years previously will not be allowed.
7. Swallowing difficulties may preclude safe use of IMT capsules.
8. Administration of IMT within 3 months prior to enrolment (probiotic administration prior to enrolment is allowed but should be recorded at screening).
9. Patients taking probiotics after enrolment to the trial.
10. Gastrointestinal disorders and diseases include delayed gastric emptying, coeliac disease, cystic fibrosis, inflammatory bowel disease, irritable bowel syndrome, chronic diarrhoea and colonic perforation or fistula.
11. Any autoimmune disease requiring, or that may require, systemic treatment with steroids and/or other immunosuppressants/immunomodulators.
12. Significant bleeding disorder (ALL, AML, AL of ambiguous lineage, MDS, CMML and CML satisfying inclusion criteria are not excluded).
13. Anaphylactic food allergy.
14. Requirement for vasopressors.
15. Valvular heart disease or known structural defects of the heart.
16. Known severe allergy to capsule components.

### Interventions

#### Allocation

50 adult patients will be allocated 1:1 between two groups:

- Capsulised IMT—as a single oral dose of 10 capsules of EBX-102-02. EBX-102-02 is encased within an intrinsically enteric-resistant capsule containing pooled, dried, full-spectrum microbial ecosystems obtained from rigorously screened donors. EBX-102-02 is characterised by the absence of pathogens, a minimum viable count of anaerobic micro-organisms and the presence of preidentified genera (such as *Faecalibacterium*), all measured by proprietary nucleic acid-based assays and other technologies. EBX-102-02 will be administered within 2 weeks of the initial study screening visit. Given the immunosuppressed nature of the recipients, out of an abundance of caution, EBX-102-02 will be prepared from CMV-negative donors, as per suggestions from current guidelines.^9^
- Matched capsulised placebo—containing microcrystalline cellulose and magnesium stearate; administered at the same point as capsulised IMT.

Treatment with either IMT or placebo will take place at 14 (±2) days prior to haematopoietic cell infusion in a hospital setting. Both IMT and placebo will be stored in a refrigerator (at 2°C–8°C) until administration, with temperature monitoring of the investigational medicinal product prior to administration. Study participants will be nil by mouth for at least 30 min prior to—and 1 hour after—each course of IMT/placebo capsule administration. They will be asked to take each capsule with sips of water and will be monitored for at least 15 min after capsule administration for complications (eg, nausea).

Randomisation will be performed centrally by the Imperial College Trials Unit (ICTU)—Cancer using OpenClinica electronic data capture system. The system applies stratified randomisation to reduce relevant imbalances and increase statistical power, randomisation will be stratified by disease history (either (1) patients known to have intestinal colonisation or bloodborne infection with MDROs during previous therapy or (2) patients without this history) to ensure there is a balanced distribution across treatment arms. To reduce predictability in the randomisation sequence, blocks of multiple sizes have been used during sequence generation.

The allocation sequence will be generated using a computerised algorithm on the Sealed Envelope system designed to maintain allocation concealment and integrity. The study uses kit codes which are pregenerated by the drug manufacturer, the kit code is linked to the treatment allocation sequence but does not reveal treatment assignment (capsule IMT or placebo). The kit codes are randomly assigned to participants through the Sealed Envelope system when randomisation is initiated in OpenClinica.

The system ensures that the allocation sequence remains concealed until the end of the study. User restrictions are in place to maintain the blinding; only personnel with distributor access can view the unblinded code lists. Study investigators and those enrolling participants cannot access these lists to preserve the double-blinded nature of the study. This ensures that the treatment assignments are hidden to both participants and investigators until the end of the study.

#### Blinding

Since this is a double-blind randomised placebo-controlled clinical trial, the treatment allocation will be blinded to the investigators, sponsor clinical trial management team, clinical staff, laboratory staff and the study participan. Placebo capsules will be identical in appearance, weight and all other obvious characteristics to the course of IMT and will be handled by pharmacy identically; this will help in maintaining blinding. Trial randomisation will occur as soon as possible after satisfactory review and confirmation of patient eligibility at screening.

Unblinding will only be considered in cases where the identity of the drug assignment is necessary for the safety of the patient. This will be possible 24 hours a day and 365 days a year, but with strong recommendation that the chief investigator and/or sponsor be contacted prior to the unblinding of the patient to discuss the reasons for unblinding. Where unblinding is required, sites will use the unique login provided by the sponsor to access the treatment assignment; if the database cannot be accessed, there will be a manual unblinding procedure in place using unblinding cards located in the pharmacy folder.

### Outcomes

The schedule/summary of visits is shown in online supplemental material 1, appendix 4, with a summary of assessments to be undertaken given in online supplemental material 1, appendix 5 [Supplementary-material SP1][Supplementary-material SP1][Supplementary-material SP1].

The primary outcome of the trial is the ability of the capsulised IMT given pre-HCT to increase and maintain intestinal microbiota diversity post-HCT. This will be assessed via measurement of the difference between the change in alpha diversity (calculated using inverse Simpson index) 28±3 days post-HCT from baseline for patients in the capsulised IMT groups versus the capsulised placebo group. The secondary objectives of the study relate to feasibility/tolerability of the capsule, and impact of the IMT on a range of clinically and translationally pertinent outcomes. These include quality of life; microbiological/infective outcomes; need for intensive care and haematological outcomes, ranging from relapse, to GvHD, to impact on engraftment and immune reconstitution. Microbiological/infective outcomes will be assessed via conventional clinical microbiology techniques, as well as via gut microbiome diversity and taxonomic characterisation. These secondary outcomes are summarised in table 1. In addition, the study has a range of discovery phase/exploratory endpoints, including investigating the impact of IMT on: markers of gut barrier function; metabolomic profiles in different biofluids (namely stool, urine and plasma); circulating cytokines; and functionality of circulating monocytes and T cells. In addition to lymphocyte subset characterisation, collection of peripheral blood mononuclear cell at day 100 (visit 8) and day 365 (visit 10) will allow further exploration of the impact of IMT on immune reconstitution and in particular, T-cell repertoire. Further details about biosample collection, storage and processing are given in online supplemental material 1, appendix 6.

**Table 1:** Secondary objectives/outcomes from the MAST study

Objectives	Outcome
Determine the feasibility and tolerability of capsule IMT prior to HCT in a multicentre setting.	Tolerability and acceptability of IMT/placebo (as assessed via patient perspective questionnaires, ie, EQ-5D-5L and EORTC QLQ-C30 questionnaires).
Evaluate microbiological/infective, haematological, and quality of life-related clinical outcomes of administering IMT prior to HCT.	Gut microbiome endpoints: Assessment of changes in inverse Simpson’s index and other measures of gut microbiome diversity across all time points assessed, including alpha diversity and richness (ie, as measured via Chao-1, Shannon, Faith’s PD) and beta-diversity (Aitchinson’s distance). Assessment of changes in gut microbiome taxonomic composition across all time points assessed(using shallow shotgun sequencing).
	Clinical endpoints: Markers of general health across all time points measured, including days on the intensive treatment unit; presence and severity of mucositis; use of (and length of time that requiring) parenteral nutrition; severe acute kidney injury and severe liver dysfunction. Infective/microbiological outcomes across all time points measured, including days of fever post-HCT (corrected for length of admission); days on antibiotics (including use of carbapenem specifically); number and length of bloodstream infections; urinary tract infections; colonisation with multidrug-resistant bacteria (including extended-spectrum beta-lactamases, vancomycin-resistant enterococci and carbapenemase-producing Enterobacteriales) and use of antibiotics. Haematological outcomes across all time points measured, including: non-relapse mortality, relapse incidence; occurrence and severity of graft-versus-host disease (GvHD), overall and GvHD-free relapse-free survival and quality of life.
Explore the potential for pre-HCT IMT to impact on HCT engraftment and immune reconstitution.	Neutrophil and platelet engraftment data as defined by EBMT will be routinely collected. Recovery of T-cell chimerism, T-cell count assessed by the lymphocyte subset analysis and immunoglobulin levels will be recorded at follow-up assessments.

EBMTEuropean Society for Blood and Marrow TransplantationEORTC QLQ-C30European Organisation for Research and Treatment of Cancer Core Quality of Life Questionnaire 30EQ-5D-5LEuroQol-5 descriptive-5 levelHCThaematopoietic cell transplantationIMTintestinal microbiota transplantMASTMicrobiota Transplant Prior to Allogeneic Stem Cell Transplant

**Figure 1:**
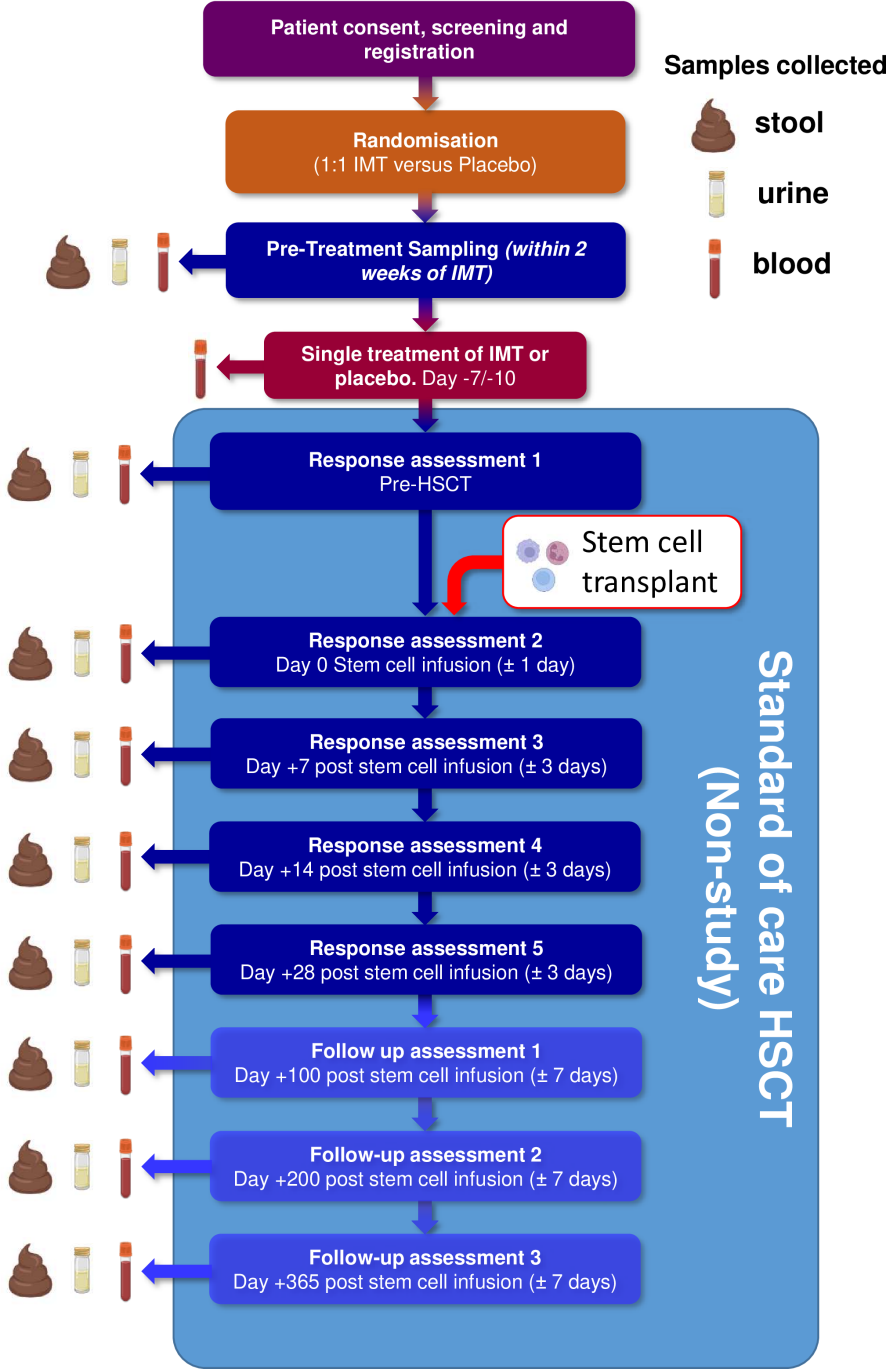
Study flow chart participant time line. HSCT, haematopoietic cell transplantation; IMT, intestinal microbiota transplant.

The study flow chart/participant timeline is shown in figure 1. Of note, regardless of whether the patient is randomised to capsulised IMT or placebo, they will continue with their scheduled standard of care treatments/assessments while also receiving study follow-up assessments at planned intervals, as shown in figure 1. Other pre-HCT and post-HCT care will be in accordance with the participating centres’ policies. As such, patients are allowed to receive prophylactic antibiotics (such as ciprofloxacin) but should not receive broad-spectrum antibiotics after the trial treatment has taken place and prior to the start of HCT. It is recognised that this may not be always possible, as neutropenic fever may sometimes develop during the conditioning therapy. If this happens, patients will not be excluded from the trial, but the broad-spectrum antibiotic use and its duration must be documented at response assessments.

### Data collection and management

#### General approach

Case report forms (CRFs) for the study will be in English, using generic names for concomitant medications wherever possible. All written material to be used by participants must use vocabulary that is clearly understood and be in the language appropriate for the study site. The electronic CRF (eCRF) database will be in OpenClinica. The investigator (or delegated member of the site study team) will record all data relating to protocol assessments and procedures, laboratory, safety and efficacy data in the eCRF. All trial documentation, including that held at participating sites and the trial coordinating centre, will be archived for a minimum of 20 years following the end of the study.

#### Confidentiality

The investigator will ensure that the participant’s confidentiality is maintained. On the CRF or other documents submitted to the sponsors, participants will be identified by a participant ID number only. Documents that are not submitted to the Sponsor (eg, signed informed consent form) should be kept in a strictly confidential file by the investigator. The investigator shall permit direct access to participants’ records and source documents for the purposes of monitoring, auditing, or inspection by the sponsor, authorised representatives of the sponsor, NHS (National Health Service), Regulatory Authorities and RECs.

The investigators and study site staff will comply with the requirements of the Data Protection Act 2018 concerning the collection, storage, processing and disclosure of personal information and will uphold the Act’s core principles.

#### Oversight and monitoring

A trial steering committee (TSC) will be convened, including as a minimum an independent chair, independent clinician, the chief investigator, independent statistician, trial manager and PPI representative. The role of the TSC is to provide overall supervision of trial conduct and progress. A trial management group (TMG) will also be convened, including the chief investigator, coinvestigators and key collaborators, trial statistician and trial manager. The TMG will be responsible for the day-to-day conduct of the trial and operational issues. Furthermore, an independent data monitoring committee (IDMC) will be convened to include as a minimum an independent oncologist chair, an independent oncologist and an independent statistician. The role of the IDMC is advisory to the TSC, to ensure the highest standard of patient safety and data integrity.

The IDMC may consider recommending the discontinuation of the trial if the recruitment rate or data quality are unacceptable, or if any issues are identified that may compromise patient safety. In the case of early discontinuation of the study, response assessments will be completed for each participant, as far as possible.

The study will be monitored periodically by trial monitors to assess the progress of the study, verify adherence to the protocol, International Conference on Harmonisation for Good Clinical Practice (ICH GCP) E6 guidelines and other national/international requirements and to review the completeness, accuracy and consistency of the data. Monitoring will be conducted centrally/remotely from the coordination centre and on-site. Monitoring procedures and requirements will be documented in a monitoring plan, developed in accordance with the risk assessment.

Quality control will be performed according to ICTU internal procedures. The study may be audited by a quality assurance representative of the sponsor and/or ICTU. All necessary data and documents will be made available for inspection.

The study may be a participant to inspection and audit by regulatory bodies to ensure adherence to GCP and the NHS Research Governance Framework for Health and Social Care (second Edition).

### Statistical considerations

#### Sample size and powering

Currently published data on alpha diversity change in patients undergoing HCT and/or IMT span different study types, vary in quality/granularity, and use different alpha diversity indices. The evidence available suggests larger decreases in alpha diversity at approximately 1-month post-HCT compared with baseline in patients who do not receive IMT relative to those that do.^2 6 8 18 19^ Fitting a mixed-effects model (with fixed effects for arm, time (day), their corresponding interaction and a random per-patient intercept effect) with quadratic splines at 5 df (3 internal knots) to longitudinal change in alpha diversity data (measured with inverse Simpson’s index) from baseline,^2^ IMT patients had an expected change in baseline alpha diversity at day 28 post-HCT 3.46 (pooled SE=2.19) units more than placebo (IMT mean change=−4.70, SE=1.44, n=14; placebo mean change=−8.16, SE=1.66, n=11). We have used these results on IMT post-HCT to design our study of IMT pre-HCT versus placebo.

Our null hypothesis is there is no difference between the change in days 28±3 days alpha diversity (inverse Simpson’s) post-HCT from baseline in patients receiving pre-HCT IMT compared with patients receiving placebo capsules (ie, the difference in between-arm changes from baseline is zero). Using a two-sample t-test to compare IMT-arm change to placebo-arm change with two-sided alpha controlled at 20%,^20 21^ we need 46 patients randomised 1:1 between IMT and placebo (23 per arm) to have ≥80% power to detect a between-arm difference of 3.46 units (with pooled SD of 5.45 estimated at day 28 post-HCT from mixed-effects model). To account for dropouts at a rate of up to 8% across both arms, we will recruit 50 patients in total. Modelling and sample size calculations have been performed using R V.3.6.1.

#### Statistical analysis

##### Overall approach

Statistical analyses will be formally documented within a detailed statistical analysis plan (SAP) and structured using the estimand framework (as described in the ICH E9 (R1) addendum on estimands and sensitivity analysis in clinical trials^21 22^ with intercurrent events and subsequent analysis strategies defined accordingly. Protocol non-adherence and other non-defined intercurrent events will be incorporated into analysis via a treatment policy where the patient is assessed based on their randomised arm regardless of the event in question. Any deviations from the SAP will be documented and signed off by the statisticians and CI and filed in the Trial Master File.

##### Primary estimand

Population: Adults with ALL, AML, AL of ambiguous lineage, high-risk MDS, CMML, and CML in blast phase, considered suitable/fit for allogeneic HCT.

Treatment: Capsulised IMT versus matched capsulised placebo.

Variable: Stool microbiota diversity post-HCT is defined as the change in alpha diversity (measured as inverse Simpson’s index) between IMT administration(at 14±3 days prior to HCT) and 28±3 days after HCT.

Population-level summary: The model-produced estimate for the treatment×time interaction effect at day 28.

Intercurrent events: Death, adverse/serious adverse events (AEs/SAEs), rescue therapy outside of antibiotic use, loss to follow-up/withdrawal.

Strategy to handle intercurrent events: Treatment policy will be used to handle patient all defined intercurrent events, any patient response-assessment data collected postrandomisation will still be used in the analysis model if day 28 data is unavailable.

##### Secondary estimand (of primary outcome)

A complete-case approach will be undertaken on the primary outcome(taking patients that provide primary outcome data across all response-assessment time points)

Population: As primary estimand.

Treatment: As primary estimand.

Variable: As primary estimand.

Population-level summary: As per primary estimand.

Intercurrent events: Death, AE/SAEs, rescue therapy outside of antibiotic use, loss to follow-up/withdrawal.

Strategy to handle intercurrent events: Treatment policy will be used in the event of AEs or rescue medication. In the event of death, withdrawal or loss to follow-up a principal stratum strategy is to be followed such that only those that complete the assessment period are to be included for analysis.

##### Secondary estimands

(1) Assessment of tolerability and acceptability of treatment versus placebo through the Functional Assessment of Cancer Therapy measured using health-related quality of life and patient perspective questionnaires.

Population: As per primary estimand.

Treatment: As per primary estimand.

Variable: Scores arising from health-related quality of life EQ-5D-5L (EuroQol-5 descriptive-5 level) and EORTC patient perspective (EORTC_QLQ-C30) questionnaires.

Population-level summary: The model-produced estimate for the treatment×time interaction effect at day 28, plus at follow-up time points days 100, day 200 and day 365.

Intercurrent events: Death, AE/SAEs, rescue therapy outside of antibiotic use, loss to follow-up/withdrawal.

Strategy to handle intercurrent events: Treatment policy as primary estimand

(2) Assessment of changes in inverse Simpson’s index and other measures of gut microbiome diversity across all time points assessed, including alpha diversity and richness (ie, as measured via Chao-1, Shannon, Faith’s PD) and beta-diversity (Aitchinson’s distance) as well as changes in gut microbiome taxonomic composition.

Population: As per primary estimand.

Variable(s): As per primary, plus Chao-1, Shannon, Faith’s PD, Aitchinson’s distance and taxonomic composition.

Population-level summary: The model-produced estimate for the treatment×time interaction effect at days 7 and 14. Interaction effect for follow-up visit time points day 100, 200 and 365 will also be assessed.

Intercurrent events: Death, AE/SAEs, rescue therapy outside of antibiotic use.

Strategy to handle intercurrent events: Treatment policy as primary estimand.

(3) Clinical endpoints including markers of general health, infective/microbiological and haematological outcomes across all time points measured, including admission to intensive care unit, survival, non-relapse mortality, relapse incidence; occurrence and severity of GvHD, overall and GvHD-free relapse-free survival and quality of life.

Population: As per primary estimand.

Variable(s): Overall and GvHD-free relapse-free survival.

Population-level summary: Log-rank test statistic, HR (with 95% CI).

Intercurrent events: Death unrelated to patient comorbidity (relapse-free survival only), death related to patient comorbidity (relapse-free survival only), loss to follow-up/patient withdrawal, AE/SAEs, rescue therapy outside of antibiotic use.

Strategy to handle intercurrent events: Death unrelated to patient comorbidity (relapse-free survival only) will be censored at the recorded time of death as part of a hypothetical strategy. Where death is potentially related, a composite strategy is to be considered where time of death will be taken as time of relapse. Loss to follow-up/patient withdrawal will be censored at time of last contact as part of a hypothetical strategy. Treatment policy will be used on use of rescue therapy or under any AE/SAE which does not result in the withdrawal of the patient.

Estimands for additional variables covered in secondary outcome #3 will be provided within the SAP. These include markers of general health (intensive treatment unit admission, severity of mucositis, occurrence of severe acute kidney injury (AKI), occurrence of severe liver dysfunction, use of oarenteral nutrition), infective haematological outcomes (fever occurrence, fever CTCAE grade, infection, multidrug-resistant bacterial colonisation (MDROs), antibiotic use), neutrophil and platelet engraftment data, recovery of T-cell chimerism, haematological outcomes (non-relapsed mortality, occurrence GvHD, severity of GvHD).

### Analysis of primary estimand

The primary outcome of between-arm difference in alpha diversity change from baseline at day 28 (±3 days) will be analysed using a mixed-effects model, with change in alpha diversity from baseline as an outcome, with treatment arm, time, treatment-by-time interactions and stratification variables used in randomisation included as fixed effects and also a per-patient intercept included as a random effect. The subsequent model estimate for the treatment-by-time interaction term at day 28 will be the effect of interest as per primary estimand.

### Analysis of secondary estimands

The complete-case analysis will follow the same model as defined in analysis of primary estimand (using only patients attending all visits as per estimand). Mixed-effect models incorporating a per-patient random effect alongside effects for time of assessment, and an interaction term of time-by-arm assessing changes in alpha diversity(inverse Simpson’s index, Chao-1, Shannon index, Faith’s PD) and β-diversity will provide treatment effects and 80% CIs at response assessments 1–5 and follow-up assessments 1–3 (see figure 1). Similar approaches will be used to assess changes in gut microbiome taxonomic composition based on shallow shotgun sequencing.

Overall survival (time from randomisation to death/date last seen alive) will be analysed using Kaplan-Meier methods and log-rank testing using the same stratification variables as per primary analysis model defined in analysis of primary estimand. Additional survival analysis will include non-relapse mortality and GvHD-free relapse-free survival.

### Analysis of secondary outcome measures

Additional analyses of clinical outcomes will include the number of days spent in intensive care; the presence and severity of mucositis and length of time requiring parenteral nutrition; days of fever post-HCT corrected for length of admission; days of antibiotics including carbapenem; the number and length of bloodstream infections; colonisation with multidrug-resistant bacteria, including extended-spectrum β-lactamases, vancomycin-resistant *Enterococci* and carbapenemase-producing *Enterobacteriaceae* and incidence of GvHD and relapse incidence. Neutrophil and platelet engraftment data, recovery of T-cell chimerism, haematological outcomes (non-relapsed mortality, occurrence GvHD and severity of GvHD).

Analysis will be completed via presentation of descriptive statistics or summary tables. Continuous outcomes will be assessed via the same mixed-model approach as per primary estimand. Frequency outcomes will use a negative binomial approach, adjusting for the same covariates as the primary estimand analysis model. In the event where data fails to satisfy model assumptions and transformation is not suitable, an appropriate non-parametric approach may be used in replacement.

Full details of analysis methodology are to be provided in the SAP.

### Safety analysis

Additional safety outcomes—including AEs, ARs (adverse reactions), SAE and SUSARs (suspected unexpected serious adverse reactions)—will be reported as frequencies, unadjusted participant proportions and/or rates where appropriate. Differences between arms with 95% CIs using exact methods will be produced where appropriate.

## Discussion

The increasing recognition of the contribution of the gut microbiome in patients with haematological malignancies undergoing cellular therapies, coupled with emergent data supporting IMT as a strategy to alter the microbiome, necessitates robust placebo-controlled IMT trials. Primarily, phase IIa trials such as MAST aim to fully evaluate the specific contribution that IMTs may have as part of patient treatment, and provide the launchpad for future phase III trials. We hope that associated microbiome, metabolomic and immune analyses will improve understanding of the mechanistic contribution of the gut microbiome to the clinical outcomes seen, potentially setting the stage for future novel targeted ‘microbiome therapeutics’ that avoid the drawbacks associated with IMT. While we envisage that most of our analyses will involve comparison of the dynamics of clinical and biological variables between the IMT and placebo arms, there may also be within-group exploratory analyses which provide further relevant insight as well (eg, comparison of ‘responders’ and ‘non-responders’ to FMT within the treatment arm only, looking for the impact of baseline host gut microbiome diversity and/or specific taxonomic features on the likelihood of response).

A growing body of non-randomised studies has described positive clinical signals when IMT was used in patients with haematological malignancies undergoing HCT.^8^ However, it was also noteworthy that a recent phase II randomised double-blind placebo-controlled trial,^23^ administering capsulised IMT or placebo after HCT for AML, timing this for after neutrophil recovery, failed to achieve its primary outcome, showing no statistical difference in the infection rate by 4 months post-HCT in the IMT arm compared with placebo. One fundamental difference in design between that study and our study is that in our trial, the IMT is targeted at the pre-HCT (rather than post-) HCT period. There were several reasons for us considering that this aspect of timing is particularly important, a factor that has also been introduced elsewhere.^24^ Most importantly, the published data related to the dynamics of the gut microbiome with HCT particularly demonstrate the close association between reduced gut microbiome diversity pre-HCT and future morbidity and mortality, as well as the emergence of *Enterococcus* domination within the gut microbiome within 3 weeks postallogeneic HCT as influencing poor outcome.^6 7^ Additionally, aberrant intestinal microbiome diversity is known to be associated with increased inflammatory response^25^ and biomarkers of inflammation measured pre-HCT were shown to be independent predictors of HCT outcomes.^26^

**Figure 2:**
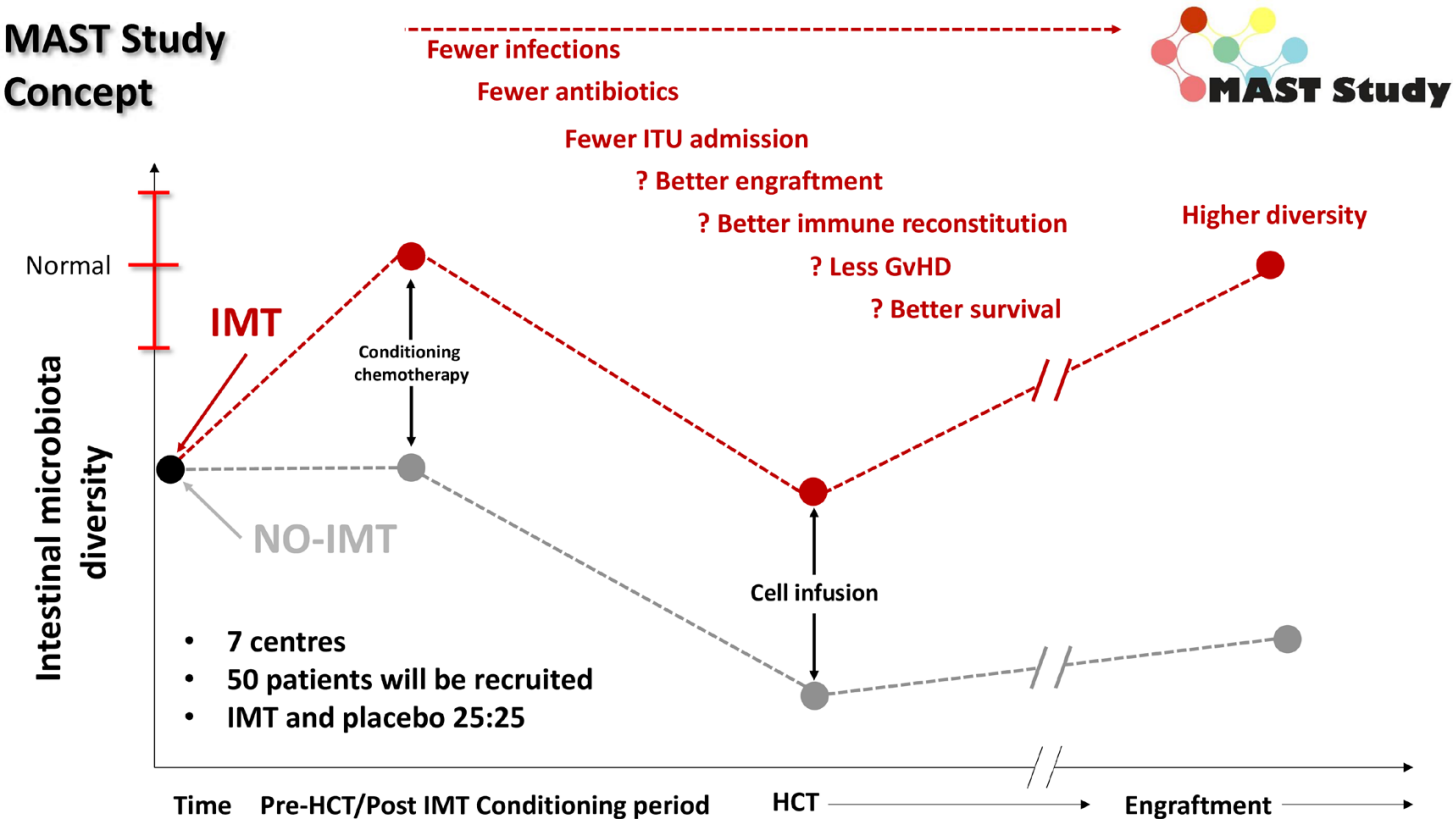
‘Prehabilitation’ of the gut microbiome in MAST. Dynamics of the gut microbiota conventionally through the peri-HCT period are shown in black (as defined previously^6^); the red line is our predicted higher starting point and nadir for patients receiving a pre-HCT IMT in the MAST trial. GvHD, graft-versus-host disease; HCT, haematopoietic cell transplantation; IMT, intestinal microbiota transplant; ITU, intensive treatment unit; MAST, Microbiota Transplant Prior to Allogeneic Stem Cell Transplant.

Therefore, we concluded that the clearest window for intervention is pre-HCT, aiming to increase the pre-HCT gut microbiota diversity, and mitigate the risk of pathobiont overgrowth is prior to start of HCT conditioning (figure 2). The concept of targeting IMT prior to intervention has also been used successfully in oncology, with a phase I study evaluating IMT use prior to immune checkpoint inhibition in 20 patients with advanced melanoma demonstrating an objective response rate of 65% (n=13/20; including 4/20 complete responses).^27^

The use of IMT in the context of immunosuppressed patients requires certain considerations above and beyond those of, for instance, conventional use of IMT in treating recurrent CDI.^28^ The use of a capsulised preparation is clearly more acceptable to this patient cohort than conventional IMT slurry and may be safer avoiding potential aspiration of slurry. The donor screening protocol used donors is in full accordance with UK recommendations,^9^ while the risk of CMV transmission via IMT appears extremely low,^29^ CMV negative donors are being used out of an abundance of caution. The window for IMT administration aims to be long enough after prior chemotherapy to allow full cell count recovery, but early enough before HCT to permit sufficient microbiota engraftment. This is important since the degree of microbiota engraftment has been associated with level of clinical improvement after IMT.^30^ Our experience to date is that IMT mitigates the risk of invasive infections related to MDROs rather than decolonises them from the gut,^15^ but there is still uncertainty regarding this; one recent trial of IMT in a renal transplant population suggested certain extended-spectrum beta-lactamases (ESBL)-colonising strains being displaced by non-ESBL strains by strain competition.^31^ The serial clinical assessment and collection of shotgun metagenomic data from study participants in a placebo-controlled fashion will allow a much more granular assessment of the impact of IMT on MDROs than has been described previously.

In conclusion, the MAST trial aspires to give new clinical and translational insights into the role of gut microbiome manipulation in patients with haematological malignancy receiving allogeneic HCT, with particular focus on the potential role of IMT on haematological and infective outcomes. The study aims to run recruitment for 24 months postauthorisation and close in May 2027.

## Ethics and dissemination

### Research ethics approval

The institutional review board (North East—Tyne & Wear South, England, Ref: 23/NE/0105) and the national regulatory authorities, Medicines & Healthcare products Regulatory Agency (MHRA, Ref: CTA 19174/0441/001-0001) issued approval on the 3 October 2023.

#### Other ethical considerations

##### Consent

Patients will be identified as per site-established processes and invited to participate by their primary haematology team. Eligible patients will be provided with the PIS (online supplemental material 2) and given sufficient time to consider the study, with opportunities to discuss and ask questions. Investigators will ensure that they adequately explain the study, including the aims, trial treatment, anticipated benefits and potential risks of participation. The right of the patient to refuse participation in the trial, or withdraw at any point, without giving explanation will be respected. Informed consent will be requested from the patient by the investigator who has been delegated the responsibility on the delegation log. Consent will be obtained no earlier than 24 hours after receiving the PIS to give them time to read and understand what their participation in the study entails (consent form provided as online supplemental material 3). With patient consent, it is the investigator’s responsibility to inform the patient’s general practitioner regarding study participation.

##### Study conduct and safety measures

The study prioritises the highest safety and ethical standards, ensuring full compliance with the 1964 Declaration of Helsinki and ICH GCP E6 guidelines. Rigorous pharmacovigilance measures are in place to monitor and reporting of serious or non-SAEs/reactions to enable a prompt and appropriate clinical response. There are stringent protocols in place for the reporting of causality, expectedness and severity assessments; every reported event undergoes thorough evaluation by clinical professionals. These safety measures are essential for maintaining participant welfare and upholding study integrity. Additionally, robust donor screenings and contraception requirements minimise potential risks associated infection transmission and unanticipated pregnancy outcomes, respectively, to reinforce a comprehensive approach to participant safety.

##### Dissemination

The dissemination of results from this study will always be performed with input from our PPI group. This will be foremost via abstract presentations at conferences and manuscripts in peer-reviewed journals. All such publications will be circulated to all authors prior to submission for their review and approval. Publications will be made in concordance with CONSORT (Consolidated Standards of Reporting Trials) guidelines/checklists. Study participants will be notified of the outcome of the trial prior to any publications. A clinical study report summarising the study results will be prepared and submitted to the research ethics committee within a year of the end of study. The results will also be submitted to the EudraCT results database in accordance with regulatory requirements.

## supplementary material

10.1136/bmjopen-2024-093120 online supplemental file 1

10.1136/bmjopen-2024-093120 online supplemental file 2

10.1136/bmjopen-2024-093120 online supplemental file 3
