# Supplementary material for: Intestinal Microbiota Transplant Prior to Allogeneic Stem Cell Transplant (MAST) trial: study protocol for a multicentre, double-blinded, placebo-controlled, phase IIa trial
Source: BMJ Open. 2024 Dec 22;14(12):e093120. doi: 10.1136/bmjopen-2024-093120 (PMC11884074; doi:10.1136/bmjopen-2024-093120)
Supplement: online supplemental file 1 [file bmjopen-14-12-s001.docx]

**Supplementary Material 1: Appendices:**

**Intestinal *M*icrobiota Transplant Prior to *A*llogeneic *S*tem Cell *T*ransplant (MAST) trial: Study Protocol for a Phase IIa Randomised Controlled Trial**

**Appendix 1 - Neutropenia inducing regimens:**

Daunorubicin and cytarabine (DA) (with or without a FLT-3 inhibitor, gemtuzumab ozogamicin, or venetoclax)

Liposomal cytarabine and daunorubicin (Vyxeos) (with or without a FLT-3 inhibitor, gemtuzumab ozogamicin, or venetoclax)

High dose cytarabine (>1000mg/m2) (with or with or without a FLT-3 inhibitor, gemtuzumab ozogamicin, or venetoclax)

Azacitine/decitabine (including oral forms) and venetoclax

Fludarabine / Cytarabine / GCSF / Idarubicin (FLAG-Ida) (with or with or without a FLT-3 inhibitor, gemtuzumab ozogamicin, venetoclax, dasatinib or ponatinib)

Clofarabine / Cytarabine / GCSF / Idarubicin (CLAG-Ida) (with or with or without a FLT-3 inhibitor, gemtuzumab ozogamicin, or venetoclax)

Mitozantrone / Etoposide / Cytarabine (MEC) (with or with or without a FLT-3 inhibitor, gemtuzumab ozogamicin, or venetoclax)

UK-ALL 14 phase 1 induction (off trial) or similar (with or without additional venetoclax)

UK-ALL 14 phase 2 induction (off trial) or similar (with or without additional venetoclax)

UK-ALL14 intensification (High dose methotrexate) or similar (with or without additional venetoclax)

Cyclophosphamide / Dexamethasone / Doxorubicin / Vincristine / Cytarabine alternating with methotrexate / cytarabine (Hyper-CVAD / MA) (with or without additional venetoclax)

**Appendix 2 – Disease and response criteria:**

***Acute lymphoblastic leukaemia (ALL) classification (International Consensus Classification of Myeloid Neoplasms and Acute Leukemias)) and response criteria (modified Center for International Blood and Marrow Transplant Research criteria)***

| **B-ALL** |
| --- |
| B-ALL with recurrent genetic abnormalities |
| B-ALL with t(9;22)(q34.1;q11.2)/*BCR::ABL1* |
| with lymphoid only involvement |
| with multilineage involvement |
| B-ALL with t(v;11q23.3)/*KMT2A* rearranged |
| B-ALL with t(12;21)(p13.2;q22.1)/*ETV6::RUNX1* |
| B-ALL, hyperdiploid |
| B-ALL, low hypodiploid |
| B-ALL, near haploid |
| B-ALL with t(5;14)(q31.1;q32.3)/*IL3::*IGH |
| B-ALL with t(1;19)(q23.3;p13.3)/*TCF3::PBX1* |
| B-ALL, *BCR*::*ABL1*–like, ABL-1 class rearranged |
| B-ALL, *BCR*::*ABL1*–like, JAK-STAT activated |
| B-ALL, *BCR*::*ABL1*–like, NOS |
| B-ALL with iAMP21 |
| B-ALL with *MYC* rearrangement |
| B-ALL with *DUX4* rearrangement |
| B-ALL with *MEF2D* rearrangement |
| B-ALL with *ZNF384(362)* rearrangement |
| B-ALL with *NUTM1* rearrangement |
| B-ALL with *HLF* rearrangement |
| B-ALL with *UBTF::ATXN7L3/PAN3,CDX2* (“CDX2/UBTF”) |
| B-ALL with mutated *IKZF1* N159Y |
| B-ALL with mutated *PAX5* P80R |
| Provisional entity: B-ALL, *ETV6::RUNX1*-like |
| Provisional entity: B-ALL, with *PAX5* alteration |
| Provisional entity: B-ALL, with mutated *ZEB2* (p.H1038R)/IGH::*CEBPE* |
| Provisional entity: B-ALL, *ZNF384* rearranged*-*like |
| Provisional entity: B-ALL, *KMT2A* rearranged-like |
| B-ALL, NOS |
| **T-ALL** |
| Early T-cell precursor ALLwith *BCL11B* rearrangement |
| Early T-cell precursor ALL, NOS |
| T-ALL, NOS |
| Provisional entities (see supplemental Table 7) |
| **Provisional entity: natural killer cell ALL** |

Complete Remission (CR)

Hematologic complete remission is defined as meeting **all** of the following response criteria for at least four weeks.

- < 5% blasts in the bone marrow
- Normal maturation of all cellular components in the bone marrow
- No extramedullary disease (e.g., CNS, soft tissue disease)
- ANC (absolute neutrophil count) ≥ 1.0 x 10^9^/L
- Platelets ≥ 100 x 10^9^/L
- Transfusion independent

In some cases, there may not be a four-week interval between completion of therapy and the pre-transplant disease assessment; in this case, CR should still be reported as the status at transplant, since it represents the “best assessment” prior to HCT. This is an exception to the criteria that CR be durable beyond four weeks. The pre-transplant disease status should not be changed based on early relapse or disease assessment post-transplant.

Include recipients who are MRD positive or where the MRD status is unknown. MRD assessments include cytogenetic, flow cytometry, and molecular methods.

Include recipients meeting the above CR criteria regardless of how many courses of therapy were required to achieve CR.

The number of this complete remission can be determined by using the following guidelines:

- 1st CR: no prior relapse
- 2nd CR: one prior relapse
- 3rd or higher: two or more prior relapses

Complete Remission with Incomplete Hematologic Recovery (CRi)

Hematologic complete remission with incomplete hematologic recovery is defined as meeting all of the following response criteria for at least four weeks:

- < 5% blasts in the bone marrow
- Normal maturation of all cellular components in the bone marrow
- No extramedullary disease (e.g., CNS, soft tissue disease)

Primary Induction Failure (PIF)

The patient received treatment for ALL but **never achieved CR or CRi at anytime**. PIF is not limited by the number of unsuccessful treatments; this disease status only applies to recipients who have never been in CR or CRi.

Relapse (REL)

Relapse is defined as the recurrence of disease after CR, meeting at least one of the following criteria:

- ≥ 5% blasts in the marrow or peripheral blood
- Extramedullary disease
- Disease presence determined by a physician upon clinical assessment

The number of this relapse can be determined by using the following guidelines:

- 1st relapse: one prior CR
- 2nd relapse: two prior CRs
- 3rd or higher: three or more CRs

Do not include a partial response (PR) when determining number of relapse. Recipients who achieve a PR to treatment should be classified as either PIF or relapse; PR in ALL is generally of short duration and is unlikely to predict clinical benefit.

| ***Acute myeloid leukaemia (AML) classification (international Consensus Classification of Myeloid Neoplasms and Acute Leukemias) and response criteria (modified Center for International Blood and Marrow Transplant Research criteria)***Acute promyelocytic leukaemia (APL) with t(15;17)(q24.1;q21.2)/*PML*::*RARA* ≥ 10% |
| --- |
| APL with other *RARA* rearrangements* ≥ 10% |
| AML with t(8;21)(q22;q22.1)/*RUNX1*::*RUNX1T1* ≥ 10% |
| AML with inv(16)(p13.1q22) or t(16;16)(p13.1;q22)/*CBFB*::*MYH11* ≥ 10% |
| AML with t(9;11)(p21.3;q23.3)/*MLLT3*::*KMT2A* ≥ 10% |
| AML with other *KMT2A* rearrangements† ≥ 10% |
| AML with t(6;9)(p22.3;q34.1)/*DEK*::*NUP214* ≥ 10% |
| AML with inv(3)(q21.3q26.2) or t(3;3)(q21.3;q26.2)/*GATA2*; *MECOM*(*EVI1*) ≥ 10% |
| AML with other *MECOM* rearrangements‡ ≥ 10% |
| AML with other rare recurring translocations (see supplemental Table 5) ≥ 10% |
| AML with t(9;22)(q34.1;q11.2)/*BCR*::*ABL1*§ ≥ 20% |
| AML with mutated *NPM1* ≥ 10% |
| AML with in-frame bZIP *CEBPA* mutations ≥ 10% |
| AML and MDS/AML with mutated *TP53*† 10-19% (MDS/AML) and ≥ 20% (AML) |
| AML and MDS/AML with myelodysplasia-related gene mutations 10-19% (MDS/AML) and ≥ 20% (AML) |
| Defined by mutations in *ASXL1*, *BCOR*, *EZH2, RUNX1*, *SF3B1, SRSF2*, *STAG2*, *U2AF1*, or *ZRSR2* |
| AML with myelodysplasia-related cytogenetic abnormalities 10-19% (MDS/AML) and ≥ 20% (AML) |
| Defined by detecting a complex karyotype (≥ 3 unrelated clonal chromosomal abnormalities in the absence of other class-defining recurring genetic abnormalities), del(5q)/t(5q)/add(5q), −7/del(7q), +8, del(12p)/t(12p)/add(12p), i(17q), −17/add(17p) or del(17p), del(20q), and/or idic(X)(q13) clonal abnormalities |
| AML not otherwise specified (NOS) 10-19% (MDS/AML) and ≥ 20% (AML) |
| Myeloid sarcoma |

*

Includes AMLs with t(1;17)(q42.3;q21.2)/*IRF2BP2*::*RARA*; t(5;17)(q35.1;q21.2)/*NPM1*::*RARA*; t(11;17)(q23.2;q21.2)/*ZBTB16*::*RARA*; cryptic inv(17q) or del(17) (q21.2q21.2)/*STAT5B*::*RARA*, *STAT3*::*RARA*; Other genes rarely rearranged with *RARA:TBL1XR1* (3q26.3), *FIP1L1* (4q12), *BCOR*(Xp11.4).

†

Includes AMLs with t(4;11)(q21.3;q23.3)/*AFF1*::*KMT2A^#^*; t(6;11)(q27;q23.3)/*AFDN*::*KMT2A*; t(10;11)(p12.3;q23.3)/*MLLT10*::*KMT2A;* t(10;11)(q21.3;q23.3)/*TET1*::*KMT2A;* t(11;19)(q23.3;p13.1)/*KMT2A*::*ELL;* t(11;19)(q23.3;p13.3)/*KMT2A*::*MLLT1* (occurs predominantly in infants and children).

‡

Includes AMLs with t(2;3)(p11∼23;q26.2)/*MECOM*::*?;* t(3;8)(q26.2;q24.2)/*MYC*, *MECOM*; t(3;12)(q26.2;p13.2)/*ETV6*::*MECOM*; t(3;21)(q26.2;q22.1)/*MECOM*::*RUNX1.*

§

The category of MDS/AML will not be used for AML with *BCR*::*ABL1* due to its overlap with progression of CML, *BCR*::*ABL1*-positive.

Complete Remission (CR)

Hematologic complete remission is defined as meeting all of the following response criteria:

- < 5% blasts in the bone marrow
- No blasts with Auer rods
- No extramedullary disease (e.g., CNS, soft tissue disease)
- Neutrophils ≥ 1.0 x 10^9^/L
- Platelets ≥ 100 x 10^9^/L
- Transfusion independent

Include recipients who are MRD positive or where the MRD status is unknown. MRD assessments include cytogenetic, flow cytometry, and molecular methods.

Include recipients meeting the above CR criteria regardless of how many courses of therapy were required to achieve CR.

The number of this complete remission can be determined by using the following guidelines:

- 1st CR: no prior relapse
- 2nd CR: one prior relapse
- 3rd or higher: two or more prior relapses

Complete Remission with Incomplete Hematologic Recovery (CRi)

Hematologic complete remission with incomplete hematologic recovery is defined as meeting all of the following response criteria:

- < 5% blasts in the bone marrow
- No blasts with Auer rods
- No extramedullary disease (e.g., CNS, soft tissue disease)

Primary Induction Failure (PIF)

The patient received treatment for AML but **never achieved CR or CRi at anytime**. PIF is not limited by the number of unsuccessful treatments; this disease status only applies to recipients who have *never been in CR or CRi*.

Relapse (REL)

Relapse is defined as the recurrence of disease after CR, meeting one or more of the following criteria:

- ≥ 5% blasts in the marrow or peripheral blood
- Extramedullary disease
- Disease presence determined by a physician upon clinical assessment

The number of this relapse can be determined by using the following guidelines:

- 1st relapse: one prior CR
- 2nd relapse: two prior CRs
- 3rd or higher: three or more CRs

Do not include a partial response (PR) when determining number of relapse. Recipients who achieve a PR to treatment should be classified as either PIF or relapse; PR in AML is generally of short duration and is unlikely to predict clinical benefit.

***Myelodysplastic syndromes (MDS) classification (International Consensus Classification of Myeloid Neoplasms and Acute Leukemias) and response criteria (modified Center for International Blood and Marrow Transplant Research criteria).***

| Myelodysplastic syndrome with mutated *SF3B1* |
| --- |
| Myelodysplastic syndrome with del(5q) |
| Myelodysplastic syndrome with mutated *TP53* |
| Myelodysplastic syndrome, not otherwise specified (MDS, NOS) |
| MDS, NOS without dysplasia |
| MDS, NOS with single lineage dysplasia |
| MDS, NOS with multilineage dysplasia |
| Myelodysplastic syndrome with excess blasts |
| Myelodysplastic syndrome/acute myeloid leukemia (MDS/AML) |
| MDS/AML with mutated *TP53* |
| MDS/AML with myelodysplasia-related gene mutations |
| MDS/AML with myelodysplasia-related cytogenetic abnormalities |
| MDS/AML, not otherwise specified |

Complete Remission (CR)

Requires all of the following maintained for a minimum of four weeks. When reporting the CR achievement date, report the first date when CR was achieved (not the four week date in which CR was maintained).

Bone marrow evaluation:

- < 5% myeloblasts with normal maturation of all cell lines

Blood evaluation

- Haemoglobin ≥ 110 g/L untransfused without erythropoietic support
- Absolute neutrophil count ≥ 1.0 x 10^9^/L without myeloid growth factor support
- Platelets ≥ 100 x 10^9^/L without thrombopoietic support
- 0% blasts in blood

In some cases, there may not be a four-week interval between completion of therapy and the pre-transplant disease assessment. In this case, CR should still be reported as the status at transplant since it represents the “best assessment” prior to HCT. This is an exception to the criteria that CR be durable beyond four weeks; the pre-transplant disease status should not be changed based on early relapse or disease assessment post-transplant.

Complete Remission with Incomplete Hematologic Recovery (CRi)

Hematologic complete remission with incomplete hematologic recovery is defined as meeting all of the following response criteria:

- < 5% blasts in the bone marrow
- No extramedullary disease (e.g., CNS, soft tissue disease)

C***hronic myeloid leukaemia with blast transformation classification (International Consensus Classification of Myeloid Neoplasms and Acute Leukemias) and response criteria***

Philadelphia positive or BCR:ABL1 CML in blast phase defined by the presence of blast ≥ 20% in blood or bone marrow.

Second chronic phase (CP2)

Requires all of the following

**Bone marrow evaluation:**

- < 5% myeloblasts with normal maturation of all cell lines

**Blood evaluation**

- Absolute neutrophil count ≥ 1.0 x 10^9^/L without myeloid growth factor support
- Platelets ≥ 100 x 10^9^/L without thrombopoietic support
- < 5% blasts in blood

**Appendix 3 – Karnofsky performance status score**

- 100 Normal; no complaints; no evidence of disease.
- 90 Able to carry on normal activity; minor signs or symptoms of disease.
- 80 Normal activity with effort; some signs or symptoms of disease.
- 70 Cares for self; unable to carry on normal activity or to do active work.
- 60 Requires occasional assistance but is able to care for most of their personal  
   needs.
- 50 Requires considerable assistance and frequent medical care.
- 40 Disabled; requires special care and assistance.
- 30 Severely disabled; hospital admission is indicated although death not  
   imminent.
- 20 Very sick; hospital admission necessary; active supportive treatment 
   necessary.
- 10 Moribund; fatal processes progressing rapidly.
- 0 Dead

**Appendix 4: Schedule/ Summary of Visits:** *Continued overleaf*

|  | **Screening** | **Treatment** | **Response Assessment:** | | | | | **Follow-up Assessment:** | | |
| --- | --- | --- | --- | --- | --- | --- | --- | --- | --- | --- |
|  |  |  | 1 | 2 | 3 | 4 | 5 | 1 | 2 | 3 |
| **Visit** | 1 | 2 | 3 | 4 | 5 | 6 | 7 | 8 | 9 | 10 |
| **Day of HCT** | From -42 | -14  (± 2 days) | -7   (± 2) | 0   (± 1) | +7   (± 3) | +14   (± 3) | +28   (± 3) | +100  (± 7) | +200  (± 7) | +365  (± 14) |
| Informed Consent | X |  |  |  |  |  |  |  |  |  |
| Inclusion & Exclusion Criteria | X |  |  |  |  |  |  |  |  |  |
| Baseline data collection/Comorbidity Index | X |  |  |  |  |  |  |  |  |  |
| Review of demographics, medical/disease | X |  |  |  |  |  |  |  |  |  |
| Pregnancy test^1^ | X |  |  |  |  |  |  |  |  |  |
| EORTC-QLQ-C30 and EQ-5D-5L Questionnaires | X |  |  |  |  |  | X | X | X | X |
| Bone marrow assessment^2^ | X |  |  |  |  |  |  | X |  | X |
| Physical Examination/Vital Signs (ECG) | <<All assessments (According to standard care practices)>> | | | | | | | | | |
| Lineage specific chimaerism^3^ |  |  |  |  |  |  |  | X | X | X |
| Lymphocyte subsets & IG levels^3^ |  |  |  |  |  |  |  | X | X | X |
| Stool Sample^7^ | X |  | X | X | X | X | X | X | X | X |
| Urine Sample^7^ | X |  | X | X | X | X | X | X | X | X |
| Blood Sample^7^ | X | X | X | X | X | X | X | X | X | X |
| Clinical data collection^5^ |  | X | X | X | X | X | X | X | X | X |
| Adverse event assessment^4^ | << Continuous assessment >> | | | | | | |  |  |  |
| Assessment of GvHD |  |  |  |  | << Continuous assessment >> | | | | | |
| Cell infusion (HCT)^6^ |  |  |  | X |  |  |  |  |  |  |

# Every effort should be made for participants to attend on the scheduled visit days. However, if a participant is unable to attend on the specified day, visits and sample collections may be arranged within the ranges as indicated above without need to report as protocol deviation.

1. Pregnancy test for women of childbearing potential: serum/urine (investigator’s discretion) pregnancy test (sensitivity of at least 25 mIU/mL) within 72 hours prior to starting study therapy. This applies even if the patient practices complete abstinence from heterosexual contact.
2. The results of bone marrow morphological, immunophenotypic, cytogenetic, and molecular characterisation performed according to local practice within the time points above should be reported within the time points above.
3. Chimaerism tests should be performed in local laboratories on day +30, +60, +90, +120, +200, and +365, lymphocyte subsets and immunoglobulin levels should be performed in local laboratories on days +100, +200, +365.
4. All AEs to be collected from written to consent to the first day of transplantation conditioning. After initiation of transplantation conditioning only AEs that are equal to or greater than Grade 3 of the CTCAE version 5.0 will be reported (unless the event meets the definition of an SAE) and abnormal laboratory findings will be reported only if they are judged to be of significant clinical importance. Reporting will stop at day +28 of transplantation. SAEs that are judged to be at least possibly related to the IMP(s) and are unexpected must still be reported in an expedited manner irrespective of how long after IMP administration the reaction occurred.
5. Collection of clinical data (see **Supplementary Material 1, Appendix 5** for summary of Assessments),

The following data will be collected at all study Visits (1-10)– Vital Signs, Physical Examination, Full Blood Count, Coagulation, Biochemistry, and Virology, Nutrition, Completed dietary questionnaire and ITU Admission. The dietary questionnaire comprises 24 hour dietary recall, a widely-used tool in studies with a nutritional component^1^; these dietary data will serve as useful metadata for the analysis of microbiome and metabolome data generated within the trial. The following data below will be collected in addition to the repeating assessments,

- Recent, Microbiology Colonisation History, Fever, Infection and Treatment History (Visits 2-10)
- Haemopoietic Cell transplant details (Visit 5 only).
- VOD, and Relapse, Engraftment, Acute GvHD, GvHD Prophylaxis and Therapy assessments (Visits 5-10).
- Post-transplant intervention assessment (Visit 10 only).

1. Haematopoietic stem cell transplant is not a study procedure and will take place as planned by the multi-disciplinary team before the patient enters the study following local standard of care procedures.
2. A summary of details for biosample collection, storage, and processing is given in **Supplementary Material 1, Appendix 6.**

**Appendix 5 - Summary of Assessments:**

|  | **Visit** | | | | | | | | | |
| --- | --- | --- | --- | --- | --- | --- | --- | --- | --- | --- |
| **Assessment Forms** | **1** | **2** | **3** | **4** | **5** | **6** | **7** | **8** | **9** | **10** |
| **Consent** | X |  |  |  |  |  |  |  |  |  |
| **Demographics** | X |  |  |  |  |  |  |  |  |  |
| **Eligibility** | X |  |  |  |  |  |  |  |  |  |
| **Significant Medical History** | X |  |  |  |  |  |  |  |  |  |
| **Chemotherapy History** | X |  |  |  |  |  |  |  |  |  |
| **Infection and Treatment History** | X |  |  |  |  |  |  |  |  |  |
| **Microbiology Colonisation History** | X |  |  |  |  |  |  |  |  |  |
| **Antibiotic History** | X |  |  |  |  |  |  |  |  |  |
| **Transplant Donor Characteristics** | X |  |  |  |  |  |  |  |  |  |
| **Comorbidity Index Score** | X |  |  |  |  |  |  |  |  |  |
| **Vital Signs** | X | X | X | X | X | X | X | X | X | X |
| **Physical Examination** | X | X | X | X | X | X | X | X | X | X |
| **Randomisation** | X |  |  |  |  |  |  |  |  |  |
| **Current Medication** | X |  |  |  |  |  |  |  |  |  |
| **Dietary Questionnaire** | X | X | X | X | X | X | X | X | X | X |
| **Full Blood Count, Coagulation, Biochemistry, and Virology** | X | X | X | X | X | X | X | X | X | X |
| **Bone Marrow Assessment Results** | X |  |  |  |  |  |  | X | X | X |
| **EQ-5D-5L Questionnaire** | X |  |  |  |  |  | X | X | X | X |
| **EOTRC QLQ-C30 Questionnaire** | X |  |  |  |  |  | X | X | X | X |
| **Sample Collection Form** |  |  | X | X | X | X | X | X | X | X |
| **Nutrition** |  | X | X | X | X | X | X | X | X | X |
| **Recent Microbiology History** |  |  | X | X | X | X | X | X | X | X |
| **Recent fever, Infection and Treatment History** |  | X | X | X | X | X | X | X | X | X |
| **Recent Microbial Colonisation History** |  | X | X | X | X | X | X | X | X | X |
| **IMP Administration** |  | X |  |  |  |  |  |  |  |  |
| **IMP Symptom Report form** |  |  | X | X | X | X | X | X | X | X |
| **Adverse Event** |  | X | X | X | X | X | X |  |  |  |
| **Haematopoietic Cell Transplant Details** |  |  |  |  | X |  |  |  |  |  |
| **VOD, and Relapse** |  |  |  |  | X | X | X | X | X | X |
| **Acute GvHD** |  |  |  |  | X | X | X | X | X | X |
| **GvHD Prophylaxis and Therapy** |  |  |  |  | X | X | X | X | X | X |
| **Engraftment** |  |  |  |  |  |  | X | X | X | X |
| **Lymphocyte Subsets and Immunoglobulin Levels - Blood** |  |  |  |  |  |  |  | X | X | X |
| **Linage Specific Chimaerisms - Blood** |  |  |  |  |  |  |  | X | X | X |
| **Chronic GVHD Assessment** |  |  |  |  |  |  |  | X | X | X |
| **Post Transplant Intervention** |  |  |  |  |  |  |  |  |  | X |
| **ITU Admissions Review** |  |  |  |  |  |  |  |  |  | X |

**Appendix 6 – Biosample collection, storage, and processing**

| Sample | Collection | Timepoints | Processing | Storage | Planned analysis |
| --- | --- | --- | --- | --- | --- |
| Stool | A faeces collection kit will be given to the study participant to provide a stool sample, which will be collected using a ’Faeces Catcher’. | All visits, except visit 2 | The study participant will scoop up the collected faecal sample and transfer this into two tubes. These are an empty faeces tube (Starstedt faeces tube), and a tube containing a preservative (Zymo DNA/ RNA Shield Fecal Collection tube). Study participant instructions for faecal collection are available at: <https://youtu.be/hG0wI5p4NKw?si=9tNMmSm-iClcot1C> . | Study participants will be given an ice pack and insulated back to transfer the collected stool in tubes from their home to the medical facility. Samples will be stored at -20°C | Shotgun sequencing, metabolomics, metaproteomics, potential future culturing. |
| Urine | Sample will be collected in a urine pot (50 ml; to collect the whole urine sample) at the medical facility. Urine should be mid-stream of the first fasted urine of the day. | All visits, except visit 2 | None | Stored at -20°C | Metabolomics. |
| Blood – plasma | Sample will be collected using 4x 3 ml lithium heparin blood tubes (i.e. 12ml of blood collected) at the medical facility. | All visits | Blood tubes will be centrifuged to pellet blood cells. Plasma will be aliquoted into 2 ml cryovials.  Blood will be processed within 2 hours, or within 24 hours stored at 4°C. | Stored at -80°C | Metabolomics, analysis for markers of gut barrier integrity. |
| Blood - PBMC | Sample will be collected using 2x 10 ml EDTA blood tubes, i.e. 20ml of blood collected) at the medical facility. | Visit 8 and 10 | PBMCs will be separated from blood by density gradient centrifugation using Ficoll. PBMCs will be washed, and a platelet removal centrifugation step will be carried out. Cells will be enumerated using a cell counter and resuspended in freezing media at 10 million cells per ml.  Blood will be processed within 24 hours. | Stored in liquid nitrogen (-196°C). | Immune reconstitution assessment by lymphocyte subsets and T-cell repertoire characterisation. |
| Blood – PaxGene RNA | Sample will be collected using 1x 2.5 ml PaxGene RNA blood tube (i.e. 2.5ml of blood collected) at the medical facility. | Visit 8 and 10 | None. Blood will be frozen within 6 hours from collection. | Stored at -80°C |  |

**References:**

1. Salvador Castell G, Serra-Majem L, Ribas-Barba L. What and how much do we eat? 24-hour dietary recall method. *Nutr Hosp* 2015;31 Suppl 3:46-8. doi: 10.3305/nh.2015.31.sup3.8750 [published Online First: 20150226]
