# Supplementary material for: Intestinal Microbiota Transplant Prior to Allogeneic Stem Cell Transplant (MAST) trial: study protocol for a multicentre, double-blinded, placebo-controlled, phase IIa trial
Source: BMJ Open. 2024 Dec 22;14(12):e093120. doi: 10.1136/bmjopen-2024-093120 (PMC11884074; doi:10.1136/bmjopen-2024-093120)
Supplement: online supplemental file 2 [file bmjopen-14-12-s002.docx]

| **MAST PATIENT INFORMATION SHEET** |
| --- |

| **Study Title** | Microbiota Transplant Prior to Allogeneic Stem Cell Transplantation (MAST) trial |
| --- | --- |
| **IRAS Project ID** | 1006971 |

# Introduction:

You are being invited to take part in a research study. Before you decide whether you wish to take part, it is important for you to understand why the research is being done and what it will involve. Someone from our team will go through the information sheet with you and answer any questions you have. Please take time to read the following information carefully and discuss it with friends, relatives, your General Practitioner (GP) and other doctors involved in your clinical care if you wish.

**Part 1** tells you the purpose of this study and what will happen to you if you take part.

**Part 2** gives you more detailed information about the conduct of the study

Please, ask us if there is anything that is not clear or if you would like more information. Take time to decide whether you wish to take part.

**Thank you for taking the time to read this information sheet.**

**Glossary of Terms**

| **Term** | **Explanation** |
| --- | --- |
| CFU | Colony forming units – Is the term used to describe the number of viable microorganisms, e.g., bacteria, there are in the capsule |
| Haematopoietic cell transplant | The clinical name for a bone marrow or blood stem cell transplant to treat blood cancer such as leukaemia. |
| Intestinal microbiota transplant | Taking stool material from a healthy donor and processing it into a capsule form for oral use. |
| Investigator | A researcher involved in a clinical study. |
| Microbiota | A collection of microorganisms that live in and on human body. |
| Microorganisms | Small organisms such as bacteria, virus particles and other single cell organisms. |
| Organism | A form of life considered as an entity, such as an animal, plant, fungus or bacterium. |
| Placebo | A substance that has no therapeutic effect, used as a control in testing new drugs |
| Plasma | The liquid part of blood that is left after all blood cells have been removed and only a clotting protein (called Fibrin) remains. |
| Phlebotomy | The procedure of drawing blood from the vein with the use of sterile material by trained and qualified healthcare personnel. |
| Sample | A small part of a substance or material obtained for testing such as blood, urine and stool/faecal material. |
| Serum | The liquid part of blood, after all, blood cells and the clotting protein (Fibrin) have been removed. |

# PART 1

# What is the purpose of this study?

Doctors and scientists have realised recently that there are billions of ‘beneficial’ bacteria and other microbes living in the human gut. These microbes do not cause us harm, but actually perform many roles in helping to keep us healthy, such as through their effects on how we process food or energy, stopping us getting infections from gut bacteria, and in how our immune system works.

When antibiotics are given to patients with blood cancers, they have a side effect of reducing the numbers of ‘beneficial bacteria’ in the gut, limiting its supportive role for the immune system. The number of ‘beneficial’ gut bacteria are important to maintain in patients who receive treatment that further impacts the immune system, such as bone marrow transplant (haematopoietic cell transplant).

The MAST clinical trial will test a way of restoration of the normal balance and diversity (range) of microbes that live in the gut prior to starting bone marrow or blood stem cell transplant (haematopoietic cell transplant). The study will also examine how this treatment affects the many complications involved in bone marrow transplantation, such as fevers (high temperatures) and infections during the transplant period. The treatment is called intestinal microbiota transplantation and involves taking bacteria from healthy people’s gut, then processing it and putting it into a capsule which when swallowed, releases the microbes into the recipient.

# Why have I been chosen?

You are being invited to take part in the research study because you will be undergoing a haematopoietic cell transplant, as part of the normal treatment for blood cancer, and because of your previous treatment (chemotherapy), you are predicted to have a lower number, and smaller range (diversity) of bacteria (microbiota) in your gut (intestines). We are looking to recruit 50 participants in total to this study. These 50 people will have acute leukaemia (AML or ALL), advanced myelodysplastic syndromes, chronic myelomonocytic leukaemia (CMML), or chronic myeloid leukaemia (CML) in blast phase and will be undergoing standard treatment (bone marrow or blood stem cell transplant) for their disease. Please read this information carefully before you decide whether to participate and ask your doctor for an explanation of anything that is not clear to you.

# Do I have to take part?

It is up to you to decide whether to take part. If you do decide to take part, you will be given this information sheet to keep and be asked to sign a consent form. If you decide to take part, you are still free to withdraw at any time and without giving a reason. A decision to withdraw at any time, or a decision not to take part, will not affect the standard of care you receive from your doctor or the hospital.

# What will happen to me if I take part?

You will be approached about entering the study before you are scheduled to have bone marrow transplantation. If you take part in this study, you will be asked to follow the study treatment plan, tests and hospital appointments for 14months). You should consider how these tests and visits will affect your work and family life and decide if you are able to commit to them.

Sometimes because we do not know which way of treating patients is best, we need to make comparisons. People will be put into groups and then compared.

The groups are selected by a computer which has no information about the individual – i.e. by chance. Subjects in each group then have a different treatment and these are compared’.

This means you have a 1 in 2 (50%) chance of receiving the treatment. Neither you nor your doctor will know which treatment group you are in (although, if your doctor needs to find out he/she can do so).

| **The study will undertake the following:**   1. Recruit 50 people with blood cancers who had treatment with chemotherapy and are about to undergo a bone marrow transplant. 2. Take 25 randomly from this group and give them the intestinal microbiota transplant using an orally taken capsule and compare them to the remaining 25 patients who will be given a placebo capsules. The capsules will be taken prior to the bone marrow transplant 3. Collect stool, blood and urine from both groups for analysis over the period of their treatment. 4. Undertake health and quality of life assessments for up to a year after their intestinal microbiota transplant and bone marrow transplant (see the schedule to the right). | 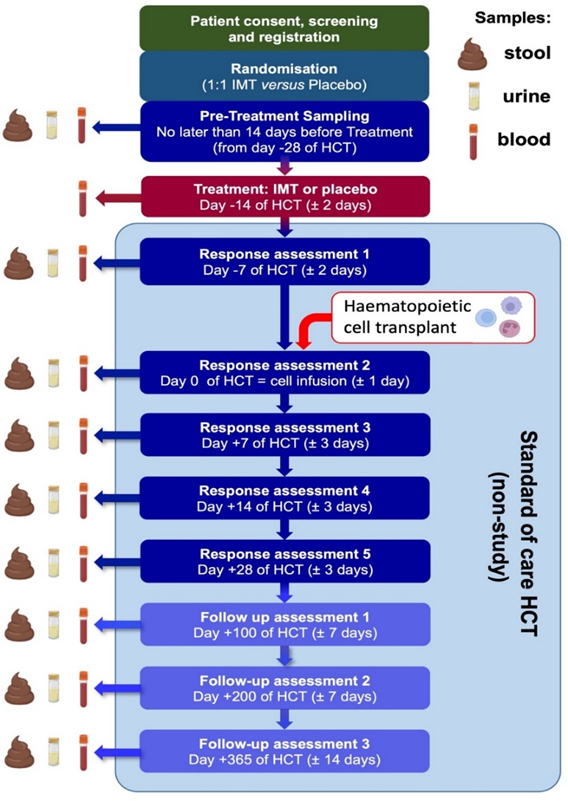 |
| --- | --- |

## What will happen before I enter the trial?

**Initial** **Study Consultation**- An initial consultation will take place to discuss participation in the study on the phone or on site with a member of our team. We will ask you some questions to see if you would be suitable to join the study, which will last approximately **15** minutes.

If we decide from the initial assessment that you are not eligible to take part in the study from the initial visit, you will unfortunately not be able to take part in the study and will continue with your planned standard of care treatment.

If we decide from the initial assessment that you may be eligible to take part in the study, we will invite you to attend a full screening visit **(Visit 1)**. We will also explain that we wish to collect a stool sample from you ahead of the next visit. We will provide you with a stool collection kit and instructions, to collect a sample at home 24 hours before or on the day of the next visit. Should you need support a family member or friend at home can help you with this or a nurse at your next study visit can help you with providing a sample.

## What happens once you are confirmed suitable to take part?

**Visit 1: Consent and Screening (within 14 days before randomisation)** – After the Initial Study Consultation, you will be invited to attend a screening visit. If you are interested in joining the study, you will be asked to sign and date the study consent form. We will perform several tests to check you are eligible for the study. All the screening tests will be explained below.

**Screening Assessments (30-60 minutes):**

A review of your medical history and any medications you are taking or have recently taken (e.g., anti-cancer treatments, over-the-counter treatments including herbal or dietary supplements, prescription medications, and/or illegal drugs)

- **A physical examination** including height and weight.
- **An assessment of your vital signs** (tests to see how well your body is functioning) including blood pressure and pulse.
- **Collect dietary information** If you have been able to give a stool sample at this visit, we will ask you to complete a dietary questionnaire to report what you have eaten 24 hours before the collection of your stool sample.
- **Quality of life Assessment** – We will ask you to complete a questionnaire to collect this information.

At this visit, we will also collect the following samples from you:

- **Blood** – A blood sample for research purposes (2 tablespoons which is 30ml) will be collected in addition to your routine blood tests.
- **Urine** – A sample kit will be given to you to provide a sample.
- **Stool** – If you are not able to provide a stool sample at this visit you will be given a self-sample kit to collect a stool sample for the next study visit.

**After we complete all the screening assessments above,**

If you are **confirmed not to be eligible** to take part in the study, you will unfortunately not be able to take part in the study and will continue with the planned standard of care treatment.

If you are **confirmed to be eligible** to take part in the study, you will randomly be assigned by a computer to one of the two treatment groups below before (within 2 weeks of Visit 2) your next scheduled study visit.

**Treatment Groups:**

**Group 1**–This is the ‘treatment arm’ of the study**:**

You will receive 10 Intestinal Microbiota Transplant(IMT) 1x10^6^ x 1x10^9^ CFU/g of viable microorganisms per oral capsule). The number of colony forming units (CFU) in each capsule may differ because the CFU of the original stool material used to make the capsules also differs. Oral capsules will be made from bacteria obtained from a healthy screened person’s stool sample; there are an extensive array of screening procedures in place to ensure the capsules are safe to take. Whilst this is not a typical ‘licensed’ medication, it has been manufactured in line with very strict approval procedures from the UK regulatory body for medicines, and this sort of treatment has already been used safely in thousands of people around the world. These capsules will be taken orally with water.

Please note – while no animal products are used in the manufacturing process, there is a possibility that the material within the capsules may contain traces of certain non-digestible dietary/ food components (for example, prawn shells); any dietary concerns you may have will be discussed with the nurse or study doctor before starting treatment.

**Group 2:**

You will receive 10 dummy oral capsules (placebo) the capsule will look the exact same as the capsule given in group one, but will contain no medicine or active ingredients. These capsules will also be taken orally with water.

**Capsule Description:**

Each capsule will be size 0 (see picture below) the capsules will be coated so that they will be able to pass through your stomach without dissolving.


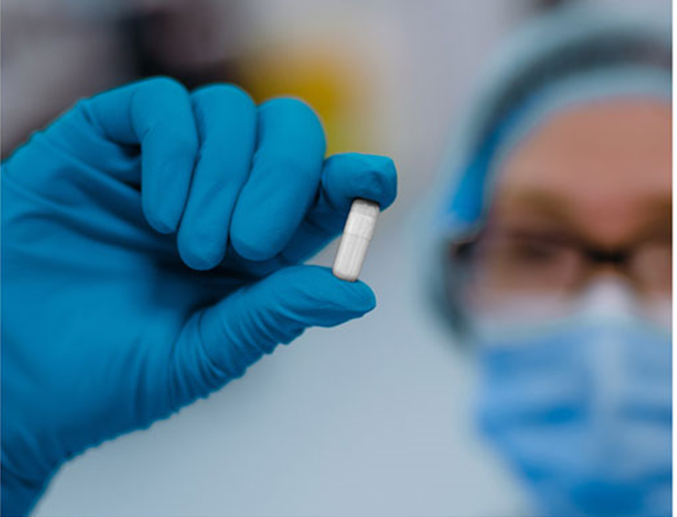


**Visit 2 (Treatment)** – Prior to this visit you will be told to avoid eating food 30 minutes before the start of this visit. You will be given either the IMT oral capsules, or the placebo oral capsules and we will also take a research blood sample and if not collected at screening urine and stool samples from you at this visit. You will receive a diary card to report daily any symptoms listed in the booklet until the next patient visit. The nurse or doctor at your next visit will go through the booklet with you should you need any support with completing the diary before the next visit, there will be a number in the booklet to use to contact a member of the study team.

After the Screening and Treatment visits have been completed, you will follow the standard of care that your clinician has discussed with you for treating the blood cancer. We will ask for you to attend **8 more visits** over the following year. These visits will occur along with your monitoring visits that you will be making as part of your treatment described in the table below.

**Study Assessments (Visit 3-10) 30-45 minutes:**

The following schedule outlines the questionnaires and samples that will be collected from you at each study visit.

| **Type of questionnaire** | **What is the questionnaire for?** | **When are they done?** |
| --- | --- | --- |
| **Health assessment questionnaire (EQ-5D-5L)** | To evaluate your general quality of life. | All Visits |
| **Quality of Life for Cancer patients (EORTC QLQ-C30)** | To evaluate your general quality of life as a cancer patient. | All Visits |
| **Dietary Questionnaire** | To understand before the stool sample was obtained, if any specific foods e.g., liquorice or fish may have been eaten. As eating certain foods can adjust the results observed during the analysis of the samples collected. | All Visits |

| **Sample type** | **What is the procedure for?** | **When are they done?** | **How will they be done?** |
| --- | --- | --- | --- |
| **Urine** | For analysis of chemicals that we think may change from before and after the intestinal microbiota transplant | All Visits | At each visit, you will be given a labelled clean container to pass urine ideally when you wake up first thing in the morning. Guidance will be provided by the examiner or qualified member of the study team. |
| **Blood** | For analysis of chemicals that we think may change from before and after the intestinal microbiota transplant | All Visits | Bloods will be collected by a qualified member of the study team |
| **Faecal** | Analysis of the microorganisms in the faecal material before and after intestinal microbiota transplantation | All Visits | We will ask you, to collect a stool sample for each visit. You can collect the sample:   - 24 hours before your next study visit a family or friend may help you with this - On the morning of each study or at the start of each visit if you would like support from a nurse.   You will be provided with a self-sampling kit that will contain:   - Clear instructions of the collection process, storage and its return - Ice pack - Bag to transport samples in |

# What do I have to do?

If you decide to take part, you will need to attend your local research centre for the assessments. If you normally require transport, we will help arrange this for you. Tests, sample collections and hospital appointments are explained in the table above, you must inform your study doctor of any medications you are currently taking or intend to take once you have entered the study.

During your participation in the MAST study, you should continue with your regular medication, and you will continue to be under the care of your regular medical team.

# How will we assess whether the treatment is working and its effect on your quality of life?

You will also be asked to complete questionnaires on paper regarding your general health and cancer usually at the same time as your scans. The questionnaires should take approximately 15-20 minutes in total to complete. If you feel uncomfortable answering any of the questions, please talk to your study doctor or nurse. You can leave blank any questions you do not want to answer. This information will help us to understand how the treatment may affect your quality of life.

# Pregnancy, contraception, and breastfeeding

If you would like to participate in this study and are a woman of childbearing potential, you must:

- Tell your study doctor immediately if you become pregnant during this study, your study doctor will advise you of the possible risks to your unborn child and discuss options for managing the pregnancy with you. If pregnancy occurs during the study, The study treatment will not be given if pregnancy occurs before the treatment visit, and you will be withdrawn from the study. If pregnancy occurs after the treatment visit you will continue attending the remaining study visits and the pregnancy will be followed until the conclusion if you give consent for this.
- Use (if you are sexually active with a male partner who has not been sterilised), one highly effective method of birth control and one additional effective barrier method of contraception at the same time. This should be done from the time of signing the informed consent form until study completion. Please discuss effective methods of contraception with your study doctor or nurse.

# What are the side effects, possible disadvantages and risks when taking part?

Being involved in a research study, such as a clinical trial, requires a degree of commitment to regular hospital visits and additional tests and surveys, and you may consider this to be a disadvantage.

The only risks associated with the study are related to some of the procedures. For example, there is minimal risk associated with blood tests, they can cause brief discomfort, bruising, or an infection in some cases, which might last for several days, and will, therefore, be performed by experienced members of the healthcare team.

Urine and stool self-collection procedures carry a minimum risk of contamination with stool and urine material; however, the risk has been minimised by the provision of an instruction manual for collection and hygiene.

There are very low risks associated with the intestinal microbiota transplantation itself and these include, fever, nausea, vomiting, bloating and constipation these should normally resolve in 1 to 2 days <https://tinyurl.com/4zpf5kch>There is also a low risk of infection from intestinal microbiota transplantation (IMT) itself. This risk is managed by EBX carrying out extensive testing on donors and their stool under supervision of a medical doctor, including blood and stool tests to detect pathogenic infectious agents, over and above as recommended by the UK experts in this field (<https://tinyurl.com/4zpf5kch>). Every stool donated, as well as every batch of IMT capsules manufactured is tested for the presence of pathogenic infectious agents and is only released for use if these are not detected.” There will be a contact number in the symptom diary card should there be any symptoms you would like to discuss the clinical team.

# What are the possible benefits of taking part?

We cannot promise the study will definitely help, however, in a small study before this larger one, we have shown that a similar treatment reduced the number of admissions to the intensive care unit, the numbers of blood infections and days of fever (high temperature) in the early days after the patients had had their bone marrow transplant. This study also showed that intestinal microbiota transplant (the treatment/method) was safe in patients undergoing bone marrow transplant, and there were no major side-effects.

The information we collect on how treatment affects the complications related to bone marrow transplantation, such as fevers (high temperatures) and infections during the transplant period may help to improve treatment and the recovery of people with blood cancers who are undergoing bone marrow transplant.

# What if I feel unhappy about continuing in the study?

If you have concerns about continuing, please discuss these with your study doctor and team. You do not have to give a reason, and your study team can explain your options to you about any data or samples collected from you as part of the study. Please see section 2 in Part 2 of the information sheet for more details of what will happen if you stop the study while on treatment.

1. What if something goes wrong?

Your study doctor will be there to answer any questions you might have regarding the cancer, its treatment, and your participation in the study. Regardless of this, if you wish to complain, or have any concerns about any aspect of the way you have been approached or treated during this study, then there will be several options available to you. Full details are included in Part 2 of this information sheet.

# Will I be compensated for taking part?

You will not be paid for taking part in the study. However, for every study visit you attend you will be able to claim back some of your expenses. You will be reimbursed up to the value of £50 (maximum £200 in total) for travel expenses per visit.

**If the information in Part 1 has interested you and you are considering taking part in the study, please read the additional information in Part 2 before making your decision.**

# PART 2

# What if new information becomes available?

Sometimes during a research project, new information or incidental findings becomes available about the treatment/drug that is being studied. If this happens, your research doctor will tell you about it and discuss with you whether you want to continue in the study. If you decide to withdraw your research doctor will make arrangements for your care to continue. If you decide to continue in the study, you will be asked to sign an updated consent form.

# What happens when the research study stops?

Once your participation is over, you will carry on with your standard treatment and medical care as usual.

Your rights to access, change or move your information are limited, as we need to manage your information in specific ways for the research to be reliable and accurate. If you withdraw from the study, we will keep the information about you that we have already obtained including any research samples unless you specifically withdraw your consent for this. At your last study visit a nurse or clinician will ask if you would like to know what treatment group you were assigned to which will be shared with you if requested by your chosen method of contact once the trial has ended.

To safeguard your rights, we will use the minimum personally identifiable information possible.

# What if there is a problem?

Imperial College London holds insurance policies which apply to this study. If you experience harm or injury because of taking part in this study, you will be eligible to claim compensation without having to prove that Imperial College London is at fault. This does not affect your legal rights to seek compensation.

If you are harmed due to someone’s negligence, you may have grounds for legal action.

Regardless of this, if you wish to complain, or have any concerns about any aspect of the way you have been treated during this study you should immediately inform the Investigator.

The normal National Health Service complaints routes are also available to you, details can be obtained from your study doctor or nurse.

If you are still not satisfied with the response, you may contact the Imperial College, Research Governance, and Integrity Team.

**Complaint’s statement**

If you wish to complain about any aspect of the way in which you have been approached or treated during this study, you should contact the study team (contact details at the end of this document) or you may contact the patient advice and liaison services (PALS) in the trust you are receiving treatment in *<Insert trust name, PALS tel.no and email>*.

# How will we use information about you?

Imperial College London is the sponsor for this study and will act as the data controller with Imperial Clinical Trials Unit – Cancer (ICTU-Ca) for this study. This means that we are responsible for looking after your information and using it appropriately. Imperial College London will keep your personal data for:

- 10 years after the study has finished in relation to data subject consent forms.
- 10 years after the study has been completed in relation to primary research data.

This study is expected to end 08/2026

We will need to use information from your medical records for this research project. This information will include your, initials, month and year of birth, gender, and ethnicity.

People within the College and study team will use this information to do the research or to check your records to make sure that the research is being done properly and the information held is accurate.

People who do not need to know who you are will not be able to see your name or contact details. Your data will have a unique code number (study ID) instead, and this code will also be used to label tissue and blood samples.

We will keep all information about you safe and secure.

Once we have finished the study, we will keep some of the data so we can check the results. We will write our reports in a way that no one can work out that you took part in the study.

**LEGAL BASIS**

As a university, we use personally-identifiable information to conduct research to improve health, care and services. As a publicly funded organisation, we have to ensure that it is in the public interest when we use personally-identifiable information from people who have agreed to take part in the research.  This means that when you agree to take part in a research study, we will use your data in the ways needed to conduct and analyse the research study. Our legal basis for using your information under the General Data Protection Regulation (GDPR) and the Data Protection Act 2018, is as follows:

Imperial College London - “performance of a task carried out in the public interest”); Health and care research should serve the public interest, which means that we have to demonstrate that our research serves the interests of society as a whole. We do this by following the [UK Policy Framework for Health and Social Care Research](https://www.hra.nhs.uk/planning-and-improving-research/policies-standards-legislation/uk-policy-framework-health-social-care-research/)

**INTERNATIONAL TRANSFERS**

There may be a requirement to transfer information to countries outside the United Kingdom (for example, to a research partner), either within the European Economic Area (EEA) or to other countries outside the EEA. Where this information contains your personal data, Imperial College London will ensure that it is transferred in accordance with data protection legislation. If the data is transferred in accordance with data protection legislation. If the data is transferred to a country which is not subject to a UK adequacy decision in respect of its data protection standards , Imperial College London will enter into a data sharing agreement with the recipient research partner that incorporates UK approved standard contractual clauses or utilise another transfer mechanism that safeguards how your personal data is processed .

You will not be able to be identified when sharing this data, but it may include demographic information such as the month and year of your birth as well as your study ID.

**SHARING YOUR INFORMATION WITH OTHERS**

We will only share your personal data with certain third parties for the purposes referred to in this participant information sheet and by relying on the legal basis for processing your data as set out below.

- Other College employees, agents, contractors and service providers (for example, suppliers of printing and mailing services, email communication services or web services, or suppliers who help us carry out any of the activities described above). Our third-party service providers are required to enter into data processing agreements with us. We only permit them to process your personal data for specified purposes and in accordance with our policies.
- EnteroBiotix Ltd., provide the capsule IMT and placebo. The following data is shared with them in this capacity as part of an agreement with Imperial College London, as well as ensuring appropriate oversight of any serious side effects that you and other study participants may experience:
  - Results data used to write reports from the study, specifically on how effective and safe the study treatment is
  - Data about serious side effect/s, including whether they got better
  - Data about medications taken and whether these were to treat the serious side effect/s or were other medications that were being taken at the time the serious side effect/s occurred.
- The Medical Research Council (MRC) who fund the study, the following data is shared with them in this capacity as part of an agreement with Imperial College London, as well as ensuring appropriate oversight of any serious side effects that you and other study participants may experience:
  - Results data used to write reports from the study, specifically on how effective and safe the study treatment is
  - Data about serious side effect/s, including whether they got better
- UK ethics and regulatory authorities who are required by law to approve and oversee research. The following data is shared with them to ensure appropriate oversight of any serious side effects that you and other study participants may experience:
  - Data about serious side effect/s, including whether they got better
  - Data about medications taken and whether these were to treat the serious side effect/s or were other medications that were being taken at the time the serious side effect/s occurred.

**POTENTIAL USE OF STUDY DATA FOR FUTURE RESEARCH**

When you agree to take part in a research study, the information collected either as part of the study or in preparation for the study (such as contact details) may, if you consent, be provided to researchers running other research studies at Imperial College London and in other organisations which may be universities or organisations involved in research in this country or abroad. Your information will only be used to conduct research in accordance with legislation including the GDPR and the UK Policy Framework for Health and Social Care Research.

This information will not identify you and will not be combined with other information in a way that could identify you, used against you or used to make decisions about you.

**COMMERCIALISATION**

Samples/data from the study may also be provided to organisations not named in this participant information sheet, e.g., commercial organisations or non-commercial organisations for the purposes of undertaking the current study, future research studies or commercial purposes such as development by a company of a new test, product or treatment. We will ensure your name and any identifying details will NOT be given to these third parties, instead you will be identified by a unique study number with any sample / data analysis having the potential to generate ‘personal data’.

Aggregated (combined) or anonymised data sets (all identifying information is removed) may also be created using your data (in a way which does not identify you individually) and be used for such research or commercial purposes where the purposes align to relevant legislation (including the GDPR) and wider aims of the study. Your data will not be shared with a commercial organisation for marketing purposes.

**WHAT ARE YOUR CHOICES ABOUT HOW YOUR INFORMATION IS USED?**

You can stop being part of the study at any time, without giving a reason, but we will keep information about you that we already have, because some research using your data may have already taken place and this cannot be undone.

- If you choose to stop taking part in the study, we would like to continue collecting information about your health from your hospital. If you do not want this to happen, tell us and we will stop. This will not affect any healthcare or support you may be receiving separately
- We need to manage your records in specific ways for the research to be reliable. This means that we won’t be able to let you see or change the data we hold about you, if this could affect the wider study or the accuracy of data collected.
- If you agree to take part in this study, you will have the option to take part in future research using your data saved from this study.

**WHERE CAN YOU FIND OUT MORE ABOUT HOW YOUR INFORMATION IS USED**

You can find out more about how we use your information

- at [www.hra.nhs.uk/information-about-patients/](https://www.hra.nhs.uk/information-about-patients/)
- by asking one of the research team
- by sending an email to [mast-trial@imperial.ac.uk](mailto:mast-trial@imperial.ac.uk)
- **COMPLAINT**

If you wish to raise a complaint on how we have handled your personal data, please contact Imperial College London’s Data Protection Officer via email at dpo@imperial.ac.uk, via telephone on 020 7594 3502 and/or via post at Imperial College London, Data Protection Officer, Faculty Building Level 4, London SW7 2AZ.

If you are not satisfied with our response or believe we are processing your personal data in a way that is not lawful you can complain to the Information Commissioner’s Office (ICO) www.ico.org.uk. The ICO does recommend that you seek to resolve matters with the data controller (us) first before involving the regulator.

# Involvement of the General Practitioner/ family doctor (GP)

With your permission, your GP and other doctors involved in your clinical care will be informed that you are taking part in this study, but otherwise all information about you and your treatment will remain strictly confidential.

# What will happen to any samples that I give?

**Blood:**

Routine blood samples will be taken and tested by your hospital as part of standard practice and destroyed immediately after testing.

Research blood samples will be sent an HTA authorised Imperial College university lab located in St Mary’s Hospital for long-term storage for future use in ethically approved studies with your permission. The blood samples will be analysed to see what chemicals they contain. Any samples left over from the process will be destroyed.

**Stool**

Stool samples will be sent to an HTA authorised Imperial College university lab located in St Mary’s Hospital for storage. Stool samples collected at your screening visit and 7^th^ visit (around 28 days after the bone marrow transplant) may be sent abroad for testing (i.e., Bacterial DNA sequencing lab in Germany) your agreement to this is required to take part in the study. The samples will be analysed to see what different types of bacteria are present in your stools. Any samples left over from the process will be destroyed.

**Urine**

The urine samples collected will be sent an HTA authorised Imperial College university lab located in St Mary’s Hospital for long-term storage for future use in ethically approved studies with your permission. They will also be analysed to see what chemicals they contain.

# What will happen to the results of the research study?

The results of the MAST study will be analysed by the MAST study research team. Results will be presented at Cancer meetings and will be published in associated journals for the wider research community to reference. No identifiable information is included in publications or presentations; therefore, you will not be identified in any report or publication. Your confidentiality is maintained throughout.

If you contact the researchers in the future, you can obtain a copy of the results.

Research data and all identifiable data will be stored by the sponsor for 10 years following the end of trial.

# Optional consent for future use of samples

If you consent, your leftover samples will be stored at our HTA authorised Imperial College Bio Bank your samples will be pseudo-anonymised and may be used for further academic and/or commercial studies by the Principal Investigator. Any such tests will have an appropriate ethical review. Upon your request at any time, your remaining samples will be destroyed.

# Who is organising and funding the research?

Imperial College London is the legal sponsor of this study and is organising the study through the Imperial Clinical Trials Unit – Cancer (ICTU-Ca). The study is funded by Medical Research Council (MRC) who will receive a study report declaring the results, but no individual research participant identifiable data will be shared. The study is organised by a research team at the Imperial Cancer Clinical Trials Unit.

The sponsor of this study will pay your hospital for including you in this study, but your doctor will not receive any personal financial payment if you take part.

# Who has reviewed the study?

All research in the NHS is looked at by an independent group of people, called a Research Ethics Committee, to protect your interests. This study has been reviewed and given a favourable opinion by <Insert name of Ethics Committee>.

# Contact for Further Information

If you have additional questions during this study about the research or your rights as a research patient, you may address them to the study doctor(s) <insert name of doctor and tel.no> or the study staff <insert name and tel. no.>. Out of office hours < insert name and tel.no> please contact the study doctor in the event of the following occurring:

a) If you suffer an illness or a possible study-related injury

b) If you feel different in any way

c) If you are admitted to the hospital for any reason

d) If you are seen at a casualty (accident/emergency department) for any reason

To speak with a member of the MAST investigator team please contact the Study Manager,

Telephone: 02075943767

Email: mast-trial@imperial.ac.uk

**Thank you for reading this information sheet. If you are interested in taking part in the study, please contact the study team to arrange a screening appointment.**

**A copy of this written information and signed Informed Consent form will be given to you.**
