# Supplementary material for: Intestinal Microbiota Transplant Prior to Allogeneic Stem Cell Transplant (MAST) trial: study protocol for a multicentre, double-blinded, placebo-controlled, phase IIa trial
Source: BMJ Open. 2024 Dec 22;14(12):e093120. doi: 10.1136/bmjopen-2024-093120 (PMC11884074; doi:10.1136/bmjopen-2024-093120)
Supplement: online supplemental file 3 [file bmjopen-14-12-s003.docx]

**Centre Number:**

**Patient Study Identification Number:**

**CONSENT FORM: MAST Study**

**Study title: M**icrobiota Transplant Prior to **A**llogeneic **S**tem Cell **T**ransplantation **(MAST)** study

**Short Title**: MAST

**Principal Investigator:** <Insert Name>

**IRAS Project ID:** 1006971

| **Consenting information** | | **Please Initial each box** |
| --- | --- | --- |
|  | I confirm that I have read and understand the Patient Information Sheet, **Version**_____, dated __________________ for the above study. I have spoken to__________________________________ and had the opportunity to consider the information, ask questions and have had these answered satisfactorily. |  |
|  | I understand that my participation is voluntary and that I am free to withdraw at any time, without providing a reason. I know that my medical care and legal rights are not affected. |  |
|  | I understand that relevant sections of my medical notes and data collected during the study may be looked at by individuals from the Sponsor (Imperial College London), from the NHS organisations, Medical Research Council, Enterobiotix or regulatory/other authorities, where it is relevant to my taking part in this research. I give permission for these individuals to have access to my records. |  |
|  | I give consent for information collected about me to be used to support other ethically approved research by an academic institution or commercial company in the future, including those outside of the United Kingdom (which Imperial has ensured will keep this information secure). |  |
|  | If Applicable, I agree to use effective contraception whilst taking part in the study, should I become pregnant after taking the study drug, I give/do not give permission for access to any of my medical notes and information collected about my pregnancy. |  |
|  | I give consent to the taking of blood **equivalent to two tablespoons** and providing urine samples for chemical analysis in this study. |  |
|  | I understand the stool collection procedure and agree to comply with these instructions. |  |
|  | I give permission for my stool samples to be sent outside of the UK for bacterial genetic analysis for this study. |  |
|  | I agree that my GP, and / or other doctors involved in my clinical care, may be notified of my participation in this study. |  |
|  | I understand that blood, urine and stool samples and / or data collected from me are a gift donated to Imperial College and that I will not personally benefit financially if this research leads to an invention and/or the successful development of a new test, medication treatment, product or service. |  |
|  | I agree to take part in the **Microbiota** Transplant Prior to **A**llogeneic **S**tem Cell **T**ransplantation **(MAST)** study |  |

| **Optional** | | **Initials** |
| --- | --- | --- |
|  | I give/do not give consent for my pseudo-anonymised stool, blood and urine sample to be stored during and at the end of the study at the University (Imperial College London) bio bank to support future ethically approved research by an academic institution or commercial company in the future, including those outside of the United Kingdom (which Imperial has ensured will keep this information secure). |  |
|  | I give permission for any pseudo-anonymised blood and urine samples to be sent outside of the UK for analysis to support future ethically approved research by an academic institution or commercial company in the future, including those outside of the United Kingdom (which Imperial has ensured will keep this information secure). |  |

Participant Name Date Signature

Name of person taking consent Date Signature

**When completed. Take 2 Copies. One to be given to the participant, one copy should be filed in the medical notes and the original stored in the Investigator Site File.**
