# Supplementary material for: Intestinal Microbiota Transplant Prior to Allogeneic Stem Cell Transplant (MAST) trial: study protocol for a multicentre, double-blinded, placebo-controlled, phase IIa trial
Source: BMJ Open. 2024 Dec 22;14(12):e093120. doi: 10.1136/bmjopen-2024-093120 (PMC11884074; doi:10.1136/bmjopen-2024-093120)
Supplement: Supplementary file 4 [file bmjopen-2024-093120.reviewer_comments.pdf]

PEER REVIEW HISTORY

BMJ Open publishes all reviews undertaken for accepted manuscripts. Reviewers are asked to complete a checklist review form and are provided with free text boxes to elaborate on their assessment. These free text comments are reproduced below.

ARTICLE DETAILS

Title (Provisional)

Intestinal Microbiota Transplant Prior to Allogeneic Stem Cell Transplant - (MAST) trial: Study Protocol for a Multi-Centre, Double-Blinded, Placebo-Controlled, Phase IIa Trial

Authors

Mullish, Benjamin; Innes, Andrew J; Roberts, Lauren A; Anim-Burton, Shian; Webber, Lee; Johnson, Nicholas Andrew; Ghani, Rohma; Farshi, Pakhshan; Khan, Anjum B; Kinsella, Francesca; Kottaridis, Panagiotis; Krishnamurthy, Pramila; Nicholson, Emma; Palanicawandar, Renuka; Wheeler, Graham; Davies, Frances; Marchesi, Julian R.; Pavlů, Jiří

VERSION 1 - REVIEW

**Reviewer**	**1**
**Name**	**Wardill, Hannah R.**
**Affiliation**	**The University of Adelaide**
**Date**	**02-Oct-2024**
**COI**	

Thank you for this exciting trial. I will watch with anticipation as it is completed.

I am especially intrigued by the pre-HSCT IMT, and agree that this has been an overlooked aspect of IMT delivery in HSCT. However, I do have some concerns regarding the durability of colonisation with the impact of chemotherapy, mucositis, empirical antibiotics, dietary changes etc that will happen after HSCT. Nonetheless, this trial is well positioned to answer this question.

My only query where additional insight would be useful related to the IMT details. You mention that 10x capsules will be used. How much stool is used to create this number of capsules? i.e. what is the microbial load? is it sufficient? Will participants take all 10x capsules at once?

Also, from the perspective of a basic scientist that has transitioned into clinical research, I really appreciate the biological primary outcome and commitment to biospecimen collection. These will enable numerous, highly informative translational studies.

Good luck with the trial.

Kind regards

Hannah Wardill (signing off as a show of my commitment to transparent review processes)

**Reviewer**	**2**
**Name**	**Ryan, Feargal**
**Affiliation**	**Flinders University**
**Date**	**07-Oct-2024**
**COI**	

Mullish et al. present a study protocol for a multi-centre phase IIa trial investigating the use of faecal microbiota transplant (FMT) prior to stem cell transplant. Overall, it is a clearly written protocol for a well-designed study, and I am excited to see the outcomes. I do, however, have a few minor concerns that can be addressed by adding some additional details which I hope will improve reproducibility:

1 - More detailed descriptions are needed regarding biosample collection, storage, and processing. As the primary outcome is related to faecal microbiota diversity, it is crucial to provide a thorough description of sample handling. The same applies to blood and urine samples. The mention of PBMC analysis is unclear (possibly flow cytometry to quantify immune cell populations? If so some details of which?). Specifics like the volume of blood/urine aimed to be collected and types of tubes used (e.g., sodium heparin, EDTA) would be helpful. Is there a time limit for processing from collection, or is a preservative/fixative being used?

2 - The dietary questionnaire mentioned needs further elaboration. Is it a food frequency questionnaire, and has it been previously validated? Additionally, how will this dietary data be incorporated into the analysis?

3 - Its possible you may end up in a situation where you have responders and non-responders in your treatment arm. If so you may want to consider having some additional secondary outcomes related to exploring that. For example using microbiota diversity changes to define a groups of responder participants which you then explore in your other data types.

4 - I did not see how sequencing data will be stored and shared. For publication, data will likely need to be deposited in a repository such as the Sequence Read Archive (SRA). Please clarify the plan for sequencing data storage and sharing, including the duration of data retention.

5 - The reviewer instructions indicate that protocol papers should include study dates. This presumably refers to the start date of sample collection and the expected end date. I couldn't see these in the current draft but please add.

6 - A brief discussion of the study's limitations might be beneficial. It could help to outline what questions this trial cannot answer.

**Reviewer**	**3**
**Name**	**Hou, Kaijian**
**Affiliation**	**Longhu People's Hospital, Endocrine Department**
**Date**	**07-Oct-2024**
**COI**	

Intestinal Microbiota Transplant Prior to Allogeneic Stem Cell Transplant - (MAST) trial: Study Protocol for a Multi-Centre, Double-Blinded, Placebo-Controlled, Phase IIa Trial. This study is to determine the ability of capsulised IMT given prior to allogeneic HCT to increase and maintain stool microbiota diversity after HCT from baseline. There are some issues in the manuscript that need to be addressed.

1. It seems that the abstract of the article does not mention the results. Should the results be reflected in the abstract?

2. Why add an article abstract or summary after a general paper abstract?

3. What are the inclusion and exclusion criteria for the study based on?

4. Has the overall logical layout of the article been reconsidered? It seems a bit strange to read?

5. Has the research conducted in the article yielded the final results?

6. Is the discussion section of the article combined with the conclusion? Does the conclusion section elaborate on the results? Is there any limitation in the explanation? I don't seem to have seen it

---

VERSION 1 - AUTHOR RESPONSE

<u>Reviewer: 1</u>

Dr. Hannah R. Wardill, The University of Adelaide

Comments to the Author:

Thank you for this exciting trial. I will watch with anticipation as it is completed. I am especially intrigued by the pre-HSCT IMT, and agree that this has been an overlooked

aspect of IMT delivery in HSCT. However, I do have some concerns regarding the durability of colonisation with the impact of chemotherapy, mucositis, empirical antibiotics, dietary changes etc that will happen after HSCT. Nonetheless, this trial is well positioned to answer this question.

Response: The authors are grateful to Dr Wardill for her feedback.

My only query where additional insight would be useful related to the IMT details. You mention that 10x capsules will be used. How much stool is used to create this number of capsules? i.e. what is the microbial load? is it sufficient? Will participants take all 10x capsules at once?

Response: While we are not able to provide some of the information requested by the Reviewer in light of its proprietary nature and due to other pending publications, we have endeavoured to expand the relevant section to include more relevant detail (page 11, lines 325-334):

- "Capsulised IMT – as a single oral dose of 10 capsules of EBX-102-02. EBX-102-02 is encased within an intrinsically enteric-resistant capsule containing pooled, dried, full-spectrum microbial ecosystems obtained from rigorously screened donors. EBX-102-02 is characterised by the absence of pathogens, a minimum viable count of anaerobic microorganisms, and the presence of particular identified genera (such as *Faecalibacterium*), all measured by proprietary nucleic acid-based assays and other technologies. EBX-102-02 will be administered within two weeks of the initial study screening visit. Given the immunosuppressed nature of the recipients, out of an abundance of caution, EBX-102-02 will be prepared from CMV-negative donors, as per suggestions from current guidelines[1]."

The dosing used in this trial was selected based on evidence from previous studies. Participants will take each capsule with water and all 10 capsules should be taken within 45 minutes.

Also, from the perspective of a basic scientist that has transitioned into clinical research, I really appreciate the biological primary outcome and commitment to biospecimen

collection. These will enable numerous, highly informative translational studies. Good luck with the trial.
Kind regards
Hannah Wardill (signing off as a show of my commitment to transparent review processes)
Response: Thank you for this feedback.

Reviewer: 2
Dr. Feargal Ryan, Flinders University
Comments to the Author:
Mullish et al. present a study protocol for a multi-centre phase IIa trial investigating the use of faecal microbiota transplant (FMT) prior to stem cell transplant. Overall, it is a clearly written protocol for a well-designed study, and I am excited to see the outcomes. I do, however, have a few minor concerns that can be addressed by adding some additional details which I hope will improve reproducibility:
Response: Thank you for the overall positive feedback.

1 - More detailed descriptions are needed regarding biosample collection, storage, and processing. As the primary outcome is related to faecal microbiota diversity, it is crucial to provide a thorough description of sample handling. The same applies to blood and urine samples. The mention of PBMC analysis is unclear (possibly flow cytometry to quantify immune cell populations? If so some details of which?). Specifics like the volume of blood/urine aimed to be collected and types of tubes used (e.g., sodium heparin, EDTA) would be helpful. Is there a time limit for processing from collection, or is a preservative/fixative being used?
Response: We agree that this is helpful, and have add a new **Supplementary Material 3, Appendix 5** file with this information, and additional specific information with regard to PBMC analysis in **Section 2.5.**

2 - The dietary questionnaire mentioned needs further elaboration. Is it a food frequency questionnaire, and has it been previously validated? Additionally, how will this dietary data be incorporated into the analysis?

Response: We agree that this is helpful, and have added further detail. In particular, we have now added the following in the legend/ notes connected to **Supplementary Material 1, Appendix 4:**

"The dietary questionnaire comprises 24 hour dietary recall, a widely-used tool in studies with a nutritional component[2]; these dietary data will serve as useful metadata for the analysis of microbiome and metabolome data generated within the trial."

3 - Its possible you may end up in a situation where you have responders and non-responders in your treatment arm. If so you may want to consider having some additional secondary outcomes related to exploring that. For example using microbiota diversity changes to define a groups of responder participants which you then explore in your other data types.

Response: This point is well-made and the specific example given very reasonable. While it is difficult to add mention of analyses such as these to the secondary outcomes (given that these have all been officially registered), we have added further discussion of this point to the **Discussion** (page 22, lines 663-668):

"Whilst we envisage that most of our analyses will involve comparison of the dynamics of clinical and biological variables between the IMT and placebo arms, there may also be within-group exploratory analyses which provide further relevant insight as well (e.g. comparison of 'responders' and 'non-responders' to FMT within the treatment arm only, looking for the impact of baseline host gut microbiome diversity and/or specific taxonomic features upon the likelihood of response)".

4 - I did not see how sequencing data will be stored and shared. For publication, data will likely need to be deposited in a repository such as the Sequence Read Archive (SRA). Please clarify the plan for sequencing data storage and sharing, including the duration of data retention.

Response: Thank you for this comment. This additional text has been added (page 29, lines 933-940):

"Regarding biological data – all such data (including sequencing, immunological and metabolomic data) will be stored locally at Imperial College London in password-controlled areas of OneDrive, SharePoint or Research Data Store as appropriate. All metagenomic sequencing data generated in this trial will be deposited at the EBI's ENA repository for public use, with all metabolomic data uploaded to the EMBL EBI MetaboLights repository; all such data will be made open access. The biological data is stored in perpetuity at the EBI's site, whilst all other trial data will be stored for 20 years after the trial has closed".

5 - The reviewer instructions indicate that protocol papers should include study dates. This presumably refers to the start date of sample collection and the expected end date. I couldn't see these in the current draft but please add.

Response: The start and end dates of the trial have been added (page 7, lines 211-212).

6 - A brief discussion of the study's limitations might be beneficial. It could help to outline what questions this trial cannot answer.

Response: Thank you; as suggested both by this Reviewer and the Editorial team, a 'Strengths and limitations of this study' section has been added.

Reviewer: 3

Dr. Kaijian Hou, Longhu People's Hospital, Shantou University Medical College

Comments to the Author:

Intestinal Microbiota Transplant Prior to Allogeneic Stem Cell Transplant - (MAST) trial: Study Protocol for a Multi-Centre, Double-Blinded, Placebo-Controlled, Phase IIa Trial. This study is to determine the ability of capsulised IMT given prior to allogeneic HCT to increase and maintain stool microbiota diversity after HCT from baseline. There are some issues in the manuscript that need to be addressed.

1. It seems that the abstract of the article does not mention the results. Should the results be reflected in the abstract?

Response: Please note that this is a protocol paper for an ongoing trial; as such, there are currently no results to report.

2. Why add an article abstract or summary after a general paper abstract?

Response: This has been amended.

3. What are the inclusion and exclusion criteria for the study based on?

Response: We included patients who are likely to have abnormal diversity of microbiota prior to haematopoietic cell transplantation (HCT), and in whom both the HCT and IMT are likely to be safe. The patient population therefore consist of patients with haematological malignancies that routinely require intensive chemotherapy followed by HCT. The vast majority of these patients normally receive broad-spectrum antibiotics during neutropenic periods following intensive chemotherapy, and we include these. We excluded patients unfit for HCT, patients with swallowing, gastroenterology, bleeding and autoimmune disorders. These exclusions were defined by us, and reviewed by the Medicines and Healthcare products Regulatory Agency (MHRA) who scrutinised the trial protocol.

4. Has the overall logical layout of the article been reconsidered? It seems a bit strange to read?

Response: In response to both this comment and Editorial feedback, there have been a number of changes of paper layout, both to try and fit 'house style' and to aid readability.

5. Has the research conducted in the article yielded the final results?

Response: Please note that this is a protocol paper for an ongoing trial; as such, there are currently no results to report.

6. Is the discussion section of the article combined with the conclusion? Does the conclusion section elaborate on the results? Is there any limitation in the explanation? I don't seem to have seen it

Response: This is an ongoing trial, and – as such - currently does not have results. As per other Reviewer and Editorial comments, a section on 'Strengths and limitations of the trial' have been added.

References:

1. Mullish BH, Merrick B, Quraishi MN, et al. The use of faecal microbiota transplant as treatment for recurrent or refractory Clostridioides difficile infection and other

potential indications: second edition of joint British Society of Gastroenterology (BSG) and Healthcare Infection Society (HIS) guidelines. *Gut* 2024;73(7):1052-75. doi: 10.1136/gutjnl-2023-331550 [published Online First: 20240606]
2. Salvador Castell G, Serra-Majem L, Ribas-Barba L. What and how much do we eat? 24-hour dietary recall method. *Nutr Hosp* 2015;31 Suppl 3:46-8. doi: 10.3305/nh.2015.31.sup3.8750 [published Online First: 20150226]

VERSION 2 - AUTHOR RESPONSE

Response to Reviewers – "bmjopen-2024-093120.R1 - "Intestinal Microbiota Transplant Prior to Allogeneic Stem Cell Transplant - (MAST) trial: Study Protocol for a Multi-Centre, Double-Blinded, Placebo-Controlled, Phase IIa Trial"

We are very grateful to the Editorial Team and Reviewers for their further considered comments regarding our manuscript. We have made some further updates and hope that the publication is now suitable for publication. Below, we have summarised those updates in turn:

*** Please revise the 'Strengths and limitations of this study' section of your manuscript (after the abstract). This section should contain up to five short bullet points, no longer than one sentence each, that relate specifically to the methods. The novelty, aims, results or expected impact of the study should not be summarised here.**

Response: Thank you; these have been revised.

*** Please provide additional detail relating to PBMC analysis as queried by reviewer 2, including volume of blood/urine aimed to be collected, types of tubes used, time limit for processing from collection**

Reviewer comment: The mention of PBMC analysis is unclear (possibly flow cytometry to quantify immune cell populations? If so some details of which?). Specifics like the volume of blood/urine aimed to be collected and types of tubes used (e.g., sodium heparin, EDTA) would be helpful. Is there a time limit for processing from collection, or is a preservative/fixative being used?

Response: We agree that this is helpful, and have add a new Supplementary Material 3, Appendix 5 file with this information, and additional specific information with regard to PBMC analysis in Section 2.5.

Response: Please note that information about the rationale for PBMC analysis is provided within the main text, which itself was added in response to prior Reviewer comments (page 13, lines 416-418):

"In addition to lymphocyte subset characterisation, collection of peripheral blood mononuclear cell (PBMC) at day 100 (visit 8), and day 365 (visit 10) will allow further exploration of the impact of IMT on immune reconstitution, and in particular, T-cell repertoire".

Furthermore, **Supplementary Material 1, Appendix 6** gives a detailed overview of biosample collection, processing and storage for this study; this has been expanded in relation to this comment to comprehensively provide the requested information. In particular, this includes detailed information about the PBMC analysis, including further information regarding the rationale.

*** Please cite Supplementary Material 1, Appendix 6 within the methods section in relation to biosample collection, storage, and processing.**

Response: This is now cited within the main text in **Section 2.5.**

Reviewer comment: More detailed descriptions are needed regarding biosample collection, storage, and processing. As the primary outcome is related to faecal microbiota diversity, it is crucial to provide a thorough description of sample handling. The same applies to blood and urine samples.

Response: We agree that this is helpful, and have add a new Supplementary Material 3, Appendix 5 file with this information

Response: We are not quite clear what the request is here; the issue may relate to us saying that this information is provided in Supplementary Material 3, Appendix 5, when it is actually in **Supplementary Material 1, Appendix 6**. We presume that this relates to more information requested about biosample collection, storage and processing; as above, we have expanded

Supplementary Material 1, Appendix 6. This also now includes a link to a YouTube clip that we are providing to study participants to inform them regarding sample collection.
